# Supplementary material for: The arch support insoles show benefits to people with flatfoot on stance time, cadence, plantar pressure and contact area
Source: PLoS One. 2020 Aug 20;15(8):e0237382. doi: 10.1371/journal.pone.0237382 (PMC7446821; doi:10.1371/journal.pone.0237382)
Supplement: S1 Data — (ZIP) [file pone.0237382.s001.zip › cadence-Interaction and main effects.docx]

|  | **Within-Subjects Factors** | | | |
| --- | --- | --- | --- | --- |
|  | Measure:MEASURE_1 | | | |
|  | insole | | slope | Dependent Variable |
| dimension1 | 1 | dimension2 | 1 | uphillASI |
|  |  |  | 2 | downhillASI |
|  |  |  | 3 | levelASI |
|  | 2 | dimension2 | 1 | uphillFI |
|  |  |  | 2 | downhillFI |
|  |  |  | 3 | levelFI |

| **Tests of Within-Subjects Effects** | | | | | | | |
| --- | --- | --- | --- | --- | --- | --- | --- |
| Measure:MEASURE_1 | | | | | | | |
| Source | | Type III Sum of Squares | df | Mean Square | F | Sig. | Partial Eta Squared |
| insole | Sphericity Assumed | 4.453 | 1 | 4.453 | 2.104 | .169 | .131 |
|  | Greenhouse-Geisser | 4.453 | 1.000 | 4.453 | 2.104 | .169 | .131 |
|  | Huynh-Feldt | 4.453 | 1.000 | 4.453 | 2.104 | .169 | .131 |
|  | Lower-bound | 4.453 | 1.000 | 4.453 | 2.104 | .169 | .131 |
| Error(insole) | Sphericity Assumed | 29.628 | 14 | 2.116 |  |  |  |
|  | Greenhouse-Geisser | 29.628 | 14.000 | 2.116 |  |  |  |
|  | Huynh-Feldt | 29.628 | 14.000 | 2.116 |  |  |  |
|  | Lower-bound | 29.628 | 14.000 | 2.116 |  |  |  |
| slope | Sphericity Assumed | 1072.996 | 2 | 536.498 | 13.206 | .000 | .485 |
|  | Greenhouse-Geisser | 1072.996 | 1.976 | 542.982 | 13.206 | .000 | .485 |
|  | Huynh-Feldt | 1072.996 | 2.000 | 536.498 | 13.206 | .000 | .485 |
|  | Lower-bound | 1072.996 | 1.000 | 1072.996 | 13.206 | .003 | .485 |
| Error(slope) | Sphericity Assumed | 1137.486 | 28 | 40.624 |  |  |  |
|  | Greenhouse-Geisser | 1137.486 | 27.666 | 41.115 |  |  |  |
|  | Huynh-Feldt | 1137.486 | 28.000 | 40.624 |  |  |  |
|  | Lower-bound | 1137.486 | 14.000 | 81.249 |  |  |  |
| insole * slope | Sphericity Assumed | 19.836 | 2 | 9.918 | 2.813 | .077 | .167 |
|  | Greenhouse-Geisser | 19.836 | 1.826 | 10.865 | 2.813 | .083 | .167 |
|  | Huynh-Feldt | 19.836 | 2.000 | 9.918 | 2.813 | .077 | .167 |
|  | Lower-bound | 19.836 | 1.000 | 19.836 | 2.813 | .116 | .167 |
| Error(insole*slope) | Sphericity Assumed | 98.709 | 28 | 3.525 |  |  |  |
|  | Greenhouse-Geisser | 98.709 | 25.559 | 3.862 |  |  |  |
|  | Huynh-Feldt | 98.709 | 28.000 | 3.525 |  |  |  |
|  | Lower-bound | 98.709 | 14.000 | 7.051 |  |  |  |

| **Pairwise Comparisons** | | | | | | | | |
| --- | --- | --- | --- | --- | --- | --- | --- | --- |
| Measure:MEASURE_1 | | | | | | | | |
| (I) slope | | (J) slope | | Mean Difference (I-J) | Std. Error | Sig.^a^ | 95% Confidence Interval for Difference^a^ | |
|  |  |  |  |  |  |  | Lower Bound | Upper Bound |
| dimension1 | 1 | dimension2 | 2 | -6.005^*^ | 1.614 | .007 | -10.391 | -1.618 |
|  |  |  | 3 | 2.156 | 1.733 | .701 | -2.553 | 6.865 |
|  | 2 | dimension2 | 1 | 6.005^*^ | 1.614 | .007 | 1.618 | 10.391 |
|  |  |  | 3 | 8.161^*^ | 1.587 | .000 | 3.848 | 12.474 |
|  | 3 | dimension2 | 1 | -2.156 | 1.733 | .701 | -6.865 | 2.553 |
|  |  |  | 2 | -8.161^*^ | 1.587 | .000 | -12.474 | -3.848 |
| Based on estimated marginal means | | | | | | | | |
| *. The mean difference is significant at the .05 level. | | | | | | | | |
| a. Adjustment for multiple comparisons: Bonferroni. | | | | | | | | |
