# Supplementary material for: The arch support insoles show benefits to people with flatfoot on stance time, cadence, plantar pressure and contact area
Source: PLoS One. 2020 Aug 20;15(8):e0237382. doi: 10.1371/journal.pone.0237382 (PMC7446821; doi:10.1371/journal.pone.0237382)
Supplement: S1 Data — (ZIP) [file pone.0237382.s001.zip › contact area-Interaction and main effects.docx]

**Forefoot :**

|  | **Within-Subjects Factors** | | | |
| --- | --- | --- | --- | --- |
|  | Measure:MEASURE_1 | | | |
|  | insole | | slope | Dependent Variable |
| dimension1 | 1 | dimension2 | 1 | FFuphillASI |
|  |  |  | 2 | FFdownhillASI |
|  |  |  | 3 | FFlevelASI |
|  | 2 | dimension2 | 1 | FFuphillFI |
|  |  |  | 2 | FFdownhillFI |
|  |  |  | 3 | FFlevelFI |

| **Descriptive Statistics** | | | |
| --- | --- | --- | --- |
|  | Mean | Std. Deviation | N |
| FFuphillASI | 41.2796 | 4.46812 | 15 |
| FFdownhillASI | 40.6054 | 5.97139 | 15 |
| FFlevelASI | 40.6230 | 3.80968 | 15 |
| FFuphillFI | 42.2439 | 5.29520 | 15 |
| FFdownhillFI | 41.8284 | 4.10555 | 15 |
| FFlevelFI | 42.3156 | 4.36763 | 15 |

| **Tests of Within-Subjects Effects** | | | | | | | |
| --- | --- | --- | --- | --- | --- | --- | --- |
| Measure:MEASURE_1 | | | | | | | |
| Source | | Type III Sum of Squares | df | Mean Square | F | Sig. | Partial Eta Squared |
| insole | Sphericity Assumed | 37.633 | 1 | 37.633 | 17.849 | .001 | .560 |
|  | Greenhouse-Geisser | 37.633 | 1.000 | 37.633 | 17.849 | .001 | .560 |
|  | Huynh-Feldt | 37.633 | 1.000 | 37.633 | 17.849 | .001 | .560 |
|  | Lower-bound | 37.633 | 1.000 | 37.633 | 17.849 | .001 | .560 |
| Error(insole) | Sphericity Assumed | 29.518 | 14 | 2.108 |  |  |  |
|  | Greenhouse-Geisser | 29.518 | 14.000 | 2.108 |  |  |  |
|  | Huynh-Feldt | 29.518 | 14.000 | 2.108 |  |  |  |
|  | Lower-bound | 29.518 | 14.000 | 2.108 |  |  |  |
| slope | Sphericity Assumed | 4.461 | 2 | 2.231 | .134 | .875 | .010 |
|  | Greenhouse-Geisser | 4.461 | 1.646 | 2.710 | .134 | .836 | .010 |
|  | Huynh-Feldt | 4.461 | 1.837 | 2.429 | .134 | .858 | .010 |
|  | Lower-bound | 4.461 | 1.000 | 4.461 | .134 | .719 | .010 |
| Error(slope) | Sphericity Assumed | 465.079 | 28 | 16.610 |  |  |  |
|  | Greenhouse-Geisser | 465.079 | 23.047 | 20.180 |  |  |  |
|  | Huynh-Feldt | 465.079 | 25.717 | 18.085 |  |  |  |
|  | Lower-bound | 465.079 | 14.000 | 33.220 |  |  |  |
| insole * slope | Sphericity Assumed | 2.045 | 2 | 1.022 | .213 | .809 | .015 |
|  | Greenhouse-Geisser | 2.045 | 1.745 | 1.172 | .213 | .780 | .015 |
|  | Huynh-Feldt | 2.045 | 1.973 | 1.037 | .213 | .807 | .015 |
|  | Lower-bound | 2.045 | 1.000 | 2.045 | .213 | .652 | .015 |
| Error(insole*slope) | Sphericity Assumed | 134.436 | 28 | 4.801 |  |  |  |
|  | Greenhouse-Geisser | 134.436 | 24.431 | 5.503 |  |  |  |
|  | Huynh-Feldt | 134.436 | 27.619 | 4.868 |  |  |  |
|  | Lower-bound | 134.436 | 14.000 | 9.603 |  |  |  |

**Midfoot:**

|  | **Within-Subjects Factors** | | | |
| --- | --- | --- | --- | --- |
|  | Measure:MEASURE_1 | | | |
|  | insole | | slope | Dependent Variable |
| dimension1 | 1 | dimension2 | 1 | MFuphillASI |
|  |  |  | 2 | MFdownhillASI |
|  |  |  | 3 | MFlevelASI |
|  | 2 | dimension2 | 1 | MFuphillFI |
|  |  |  | 2 | MFdownhillFI |
|  |  |  | 3 | MFlevelFI |

| **Descriptive Statistics** | | | |
| --- | --- | --- | --- |
|  | Mean | Std. Deviation | N |
| MFuphillASI | 30.9160 | 5.16867 | 15 |
| MFdownhillASI | 30.0390 | 5.40050 | 15 |
| MFlevelASI | 30.5389 | 4.77834 | 15 |
| MFuphillFI | 29.3587 | 6.15755 | 15 |
| MFdownhillFI | 29.2324 | 6.07813 | 15 |
| MFlevelFI | 28.9761 | 6.08019 | 15 |

| **Tests of Within-Subjects Effects** | | | | | | | |
| --- | --- | --- | --- | --- | --- | --- | --- |
| Measure:MEASURE_1 | | | | | | | |
| Source | | Type III Sum of Squares | df | Mean Square | F | Sig. | Partial Eta Squared |
| insole | Sphericity Assumed | 38.546 | 1 | 38.546 | 15.813 | .001 | .530 |
|  | Greenhouse-Geisser | 38.546 | 1.000 | 38.546 | 15.813 | .001 | .530 |
|  | Huynh-Feldt | 38.546 | 1.000 | 38.546 | 15.813 | .001 | .530 |
|  | Lower-bound | 38.546 | 1.000 | 38.546 | 15.813 | .001 | .530 |
| Error(insole) | Sphericity Assumed | 34.128 | 14 | 2.438 |  |  |  |
|  | Greenhouse-Geisser | 34.128 | 14.000 | 2.438 |  |  |  |
|  | Huynh-Feldt | 34.128 | 14.000 | 2.438 |  |  |  |
|  | Lower-bound | 34.128 | 14.000 | 2.438 |  |  |  |
| slope | Sphericity Assumed | 4.108 | 2 | 2.054 | .131 | .878 | .009 |
|  | Greenhouse-Geisser | 4.108 | 1.465 | 2.803 | .131 | .814 | .009 |
|  | Huynh-Feldt | 4.108 | 1.594 | 2.577 | .131 | .832 | .009 |
|  | Lower-bound | 4.108 | 1.000 | 4.108 | .131 | .723 | .009 |
| Error(slope) | Sphericity Assumed | 439.260 | 28 | 15.688 |  |  |  |
|  | Greenhouse-Geisser | 439.260 | 20.516 | 21.411 |  |  |  |
|  | Huynh-Feldt | 439.260 | 22.318 | 19.682 |  |  |  |
|  | Lower-bound | 439.260 | 14.000 | 31.376 |  |  |  |
| insole * slope | Sphericity Assumed | 2.838 | 2 | 1.419 | 1.127 | .338 | .075 |
|  | Greenhouse-Geisser | 2.838 | 1.650 | 1.720 | 1.127 | .331 | .075 |
|  | Huynh-Feldt | 2.838 | 1.843 | 1.540 | 1.127 | .335 | .075 |
|  | Lower-bound | 2.838 | 1.000 | 2.838 | 1.127 | .306 | .075 |
| Error(insole*slope) | Sphericity Assumed | 35.247 | 28 | 1.259 |  |  |  |
|  | Greenhouse-Geisser | 35.247 | 23.105 | 1.525 |  |  |  |
|  | Huynh-Feldt | 35.247 | 25.797 | 1.366 |  |  |  |
|  | Lower-bound | 35.247 | 14.000 | 2.518 |  |  |  |

**Heel:**

|  | **Within-Subjects Factors** | | | |
| --- | --- | --- | --- | --- |
|  | Measure:MEASURE_1 | | | |
|  | insole | | slope | Dependent Variable |
| dimension1 | 1 | dimension2 | 1 | HuphillASI |
|  |  |  | 2 | HdownhillASI |
|  |  |  | 3 | HlevelASI |
|  | 2 | dimension2 | 1 | HuphillFI |
|  |  |  | 2 | HdownhillFI |
|  |  |  | 3 | HlevelFI |

| **Descriptive Statistics** | | | |
| --- | --- | --- | --- |
|  | Mean | Std. Deviation | N |
| HuphillASI | 27.8044 | 2.27690 | 15 |
| HdownhillASI | 29.3556 | 2.25294 | 15 |
| HlevelASI | 28.8381 | 2.61747 | 15 |
| HuphillFI | 28.3974 | 2.22446 | 15 |
| HdownhillFI | 28.9393 | 2.51808 | 15 |
| HlevelFI | 28.7083 | 2.47508 | 15 |

| **Tests of Within-Subjects Effects** | | | | | | | |
| --- | --- | --- | --- | --- | --- | --- | --- |
| Measure:MEASURE_1 | | | | | | | |
| Source | | Type III Sum of Squares | df | Mean Square | F | Sig. | Partial Eta Squared |
| insole | Sphericity Assumed | .005 | 1 | .005 | .003 | .959 | .000 |
|  | Greenhouse-Geisser | .005 | 1.000 | .005 | .003 | .959 | .000 |
|  | Huynh-Feldt | .005 | 1.000 | .005 | .003 | .959 | .000 |
|  | Lower-bound | .005 | 1.000 | .005 | .003 | .959 | .000 |
| Error(insole) | Sphericity Assumed | 28.027 | 14 | 2.002 |  |  |  |
|  | Greenhouse-Geisser | 28.027 | 14.000 | 2.002 |  |  |  |
|  | Huynh-Feldt | 28.027 | 14.000 | 2.002 |  |  |  |
|  | Lower-bound | 28.027 | 14.000 | 2.002 |  |  |  |
| slope | Sphericity Assumed | 16.873 | 2 | 8.436 | 1.861 | .174 | .117 |
|  | Greenhouse-Geisser | 16.873 | 1.991 | 8.474 | 1.861 | .174 | .117 |
|  | Huynh-Feldt | 16.873 | 2.000 | 8.436 | 1.861 | .174 | .117 |
|  | Lower-bound | 16.873 | 1.000 | 16.873 | 1.861 | .194 | .117 |
| Error(slope) | Sphericity Assumed | 126.921 | 28 | 4.533 |  |  |  |
|  | Greenhouse-Geisser | 126.921 | 27.876 | 4.553 |  |  |  |
|  | Huynh-Feldt | 126.921 | 28.000 | 4.533 |  |  |  |
|  | Lower-bound | 126.921 | 14.000 | 9.066 |  |  |  |
| insole * slope | Sphericity Assumed | 4.058 | 2 | 2.029 | .540 | .589 | .037 |
|  | Greenhouse-Geisser | 4.058 | 1.419 | 2.860 | .540 | .532 | .037 |
|  | Huynh-Feldt | 4.058 | 1.533 | 2.648 | .540 | .544 | .037 |
|  | Lower-bound | 4.058 | 1.000 | 4.058 | .540 | .475 | .037 |
| Error(insole*slope) | Sphericity Assumed | 105.260 | 28 | 3.759 |  |  |  |
|  | Greenhouse-Geisser | 105.260 | 19.863 | 5.299 |  |  |  |
|  | Huynh-Feldt | 105.260 | 21.456 | 4.906 |  |  |  |
|  | Lower-bound | 105.260 | 14.000 | 7.519 |  |  |  |
