# Supplementary material for: The arch support insoles show benefits to people with flatfoot on stance time, cadence, plantar pressure and contact area
Source: PLoS One. 2020 Aug 20;15(8):e0237382. doi: 10.1371/journal.pone.0237382 (PMC7446821; doi:10.1371/journal.pone.0237382)
Supplement: S1 Data — (ZIP) [file pone.0237382.s001.zip › peak pressure-Interaction and main effects.docx]

**BT:**

|  | **Within-Subjects Factors** | | | |
| --- | --- | --- | --- | --- |
|  | Measure:MEASURE_1 | | | |
|  | insole | | slope | Dependent Variable |
| dimension1 | 1 | dimension2 | 1 | BTuphillASI |
|  |  |  | 2 | BTdownhillASI |
|  |  |  | 3 | BTlevelASI |
|  | 2 | dimension2 | 1 | BTuphillFI |
|  |  |  | 2 | BTdownhillFI |
|  |  |  | 3 | BTlevelFI |

| **Descriptive Statistics** | | | |
| --- | --- | --- | --- |
|  | Mean | Std. Deviation | N |
| BTuphillASI | 407.7000 | 249.94312 | 15 |
| BTdownhillASI | 322.7333 | 133.89373 | 15 |
| BTlevelASI | 226.4000 | 80.64610 | 15 |
| BTuphillFI | 294.1000 | 171.99171 | 15 |
| BTdownhillFI | 284.8333 | 111.10463 | 15 |
| BTlevelFI | 171.2000 | 74.67711 | 15 |

| **Tests of Within-Subjects Effects** | | | | | | | |
| --- | --- | --- | --- | --- | --- | --- | --- |
| Measure:MEASURE_1 | | | | | | | |
| Source | | Type III Sum of Squares | df | Mean Square | F | Sig. | Partial Eta Squared |
| insole | Sphericity Assumed | 106812.225 | 1 | 106812.225 | 12.335 | .003 | .468 |
|  | Greenhouse-Geisser | 106812.225 | 1.000 | 106812.225 | 12.335 | .003 | .468 |
|  | Huynh-Feldt | 106812.225 | 1.000 | 106812.225 | 12.335 | .003 | .468 |
|  | Lower-bound | 106812.225 | 1.000 | 106812.225 | 12.335 | .003 | .468 |
| Error(insole) | Sphericity Assumed | 121227.483 | 14 | 8659.106 |  |  |  |
|  | Greenhouse-Geisser | 121227.483 | 14.000 | 8659.106 |  |  |  |
|  | Huynh-Feldt | 121227.483 | 14.000 | 8659.106 |  |  |  |
|  | Lower-bound | 121227.483 | 14.000 | 8659.106 |  |  |  |
| slope | Sphericity Assumed | 363758.906 | 2 | 181879.453 | 7.342 | .003 | .344 |
|  | Greenhouse-Geisser | 363758.906 | 1.570 | 231699.980 | 7.342 | .006 | .344 |
|  | Huynh-Feldt | 363758.906 | 1.734 | 209822.537 | 7.342 | .004 | .344 |
|  | Lower-bound | 363758.906 | 1.000 | 363758.906 | 7.342 | .017 | .344 |
| Error(slope) | Sphericity Assumed | 693586.178 | 28 | 24770.935 |  |  |  |
|  | Greenhouse-Geisser | 693586.178 | 21.979 | 31556.204 |  |  |  |
|  | Huynh-Feldt | 693586.178 | 24.271 | 28576.622 |  |  |  |
|  | Lower-bound | 693586.178 | 14.000 | 49541.870 |  |  |  |
| insole * slope | Sphericity Assumed | 23600.850 | 2 | 11800.425 | 3.995 | .030 | .222 |
|  | Greenhouse-Geisser | 23600.850 | 1.875 | 12585.883 | 3.995 | .033 | .222 |
|  | Huynh-Feldt | 23600.850 | 2.000 | 11800.425 | 3.995 | .030 | .222 |
|  | Lower-bound | 23600.850 | 1.000 | 23600.850 | 3.995 | .065 | .222 |
| Error(insole*slope) | Sphericity Assumed | 82709.567 | 28 | 2953.913 |  |  |  |
|  | Greenhouse-Geisser | 82709.567 | 26.253 | 3150.531 |  |  |  |
|  | Huynh-Feldt | 82709.567 | 28.000 | 2953.913 |  |  |  |
|  | Lower-bound | 82709.567 | 14.000 | 5907.826 |  |  |  |

**M1:**

|  | **Within-Subjects Factors** | | | |
| --- | --- | --- | --- | --- |
|  | Measure:MEASURE_1 | | | |
|  | insole | | slope | Dependent Variable |
| dimension1 | 1 | dimension2 | 1 | M1uphillASI |
|  |  |  | 2 | M1downhillASI |
|  |  |  | 3 | M1levelASI |
|  | 2 | dimension2 | 1 | M1uphillFI |
|  |  |  | 2 | M1downhillFI |
|  |  |  | 3 | M1levelFI |

| **Descriptive Statistics** | | | |
| --- | --- | --- | --- |
|  | Mean | Std. Deviation | N |
| M1uphillASI | 259.6667 | 119.52909 | 15 |
| M1downhillASI | 220.2333 | 138.78823 | 15 |
| M1levelASI | 216.0667 | 93.07425 | 15 |
| M1uphillFI | 245.9333 | 96.90272 | 15 |
| M1downhillFI | 201.5333 | 109.99082 | 15 |
| M1levelFI | 205.5667 | 77.05352 | 15 |

| **Tests of Within-Subjects Effects** | | | | | | | |
| --- | --- | --- | --- | --- | --- | --- | --- |
| Measure:MEASURE_1 | | | | | | | |
| Source | | Type III Sum of Squares | df | Mean Square | F | Sig. | Partial Eta Squared |
| insole | Sphericity Assumed | 4608.178 | 1 | 4608.178 | 2.660 | .125 | .160 |
|  | Greenhouse-Geisser | 4608.178 | 1.000 | 4608.178 | 2.660 | .125 | .160 |
|  | Huynh-Feldt | 4608.178 | 1.000 | 4608.178 | 2.660 | .125 | .160 |
|  | Lower-bound | 4608.178 | 1.000 | 4608.178 | 2.660 | .125 | .160 |
| Error(insole) | Sphericity Assumed | 24254.822 | 14 | 1732.487 |  |  |  |
|  | Greenhouse-Geisser | 24254.822 | 14.000 | 1732.487 |  |  |  |
|  | Huynh-Feldt | 24254.822 | 14.000 | 1732.487 |  |  |  |
|  | Lower-bound | 24254.822 | 14.000 | 1732.487 |  |  |  |
| slope | Sphericity Assumed | 35196.117 | 2 | 17598.058 | 4.088 | .028 | .226 |
|  | Greenhouse-Geisser | 35196.117 | 1.429 | 24631.319 | 4.088 | .044 | .226 |
|  | Huynh-Feldt | 35196.117 | 1.546 | 22767.253 | 4.088 | .040 | .226 |
|  | Lower-bound | 35196.117 | 1.000 | 35196.117 | 4.088 | .063 | .226 |
| Error(slope) | Sphericity Assumed | 120521.967 | 28 | 4304.356 |  |  |  |
|  | Greenhouse-Geisser | 120521.967 | 20.005 | 6024.640 |  |  |  |
|  | Huynh-Feldt | 120521.967 | 21.643 | 5568.703 |  |  |  |
|  | Lower-bound | 120521.967 | 14.000 | 8608.712 |  |  |  |
| insole * slope | Sphericity Assumed | 255.906 | 2 | 127.953 | .119 | .888 | .008 |
|  | Greenhouse-Geisser | 255.906 | 1.621 | 157.894 | .119 | .847 | .008 |
|  | Huynh-Feldt | 255.906 | 1.802 | 141.988 | .119 | .869 | .008 |
|  | Lower-bound | 255.906 | 1.000 | 255.906 | .119 | .735 | .008 |
| Error(insole*slope) | Sphericity Assumed | 30133.844 | 28 | 1076.209 |  |  |  |
|  | Greenhouse-Geisser | 30133.844 | 22.690 | 1328.044 |  |  |  |
|  | Huynh-Feldt | 30133.844 | 25.232 | 1194.261 |  |  |  |
|  | Lower-bound | 30133.844 | 14.000 | 2152.417 |  |  |  |

**M2:**

|  | **Within-Subjects Factors** | | | |
| --- | --- | --- | --- | --- |
|  | Measure:MEASURE_1 | | | |
|  | insole | | slope | Dependent Variable |
| dimension1 | 1 | dimension2 | 1 | M2uphillASI |
|  |  |  | 2 | M2downhillASI |
|  |  |  | 3 | M2levelASI |
|  | 2 | dimension2 | 1 | M2uphillFI |
|  |  |  | 2 | M2downhillFI |
|  |  |  | 3 | M2levelFI |

| **Descriptive Statistics** | | | |
| --- | --- | --- | --- |
|  | Mean | Std. Deviation | N |
| M2uphillASI | 310.5667 | 212.15559 | 15 |
| M2downhillASI | 290.3667 | 196.41678 | 15 |
| M2levelASI | 367.2667 | 250.45158 | 15 |
| M2uphillFI | 304.0000 | 196.25657 | 15 |
| M2downhillFI | 251.2000 | 181.97937 | 15 |
| M2levelFI | 332.3000 | 196.50571 | 15 |

| **Tests of Within-Subjects Effects** | | | | | | | |
| --- | --- | --- | --- | --- | --- | --- | --- |
| Measure:MEASURE_1 | | | | | | | |
| Source | | Type III Sum of Squares | df | Mean Square | F | Sig. | Partial Eta Squared |
| insole | Sphericity Assumed | 16281.225 | 1 | 16281.225 | 5.366 | .036 | .277 |
|  | Greenhouse-Geisser | 16281.225 | 1.000 | 16281.225 | 5.366 | .036 | .277 |
|  | Huynh-Feldt | 16281.225 | 1.000 | 16281.225 | 5.366 | .036 | .277 |
|  | Lower-bound | 16281.225 | 1.000 | 16281.225 | 5.366 | .036 | .277 |
| Error(insole) | Sphericity Assumed | 42480.317 | 14 | 3034.308 |  |  |  |
|  | Greenhouse-Geisser | 42480.317 | 14.000 | 3034.308 |  |  |  |
|  | Huynh-Feldt | 42480.317 | 14.000 | 3034.308 |  |  |  |
|  | Lower-bound | 42480.317 | 14.000 | 3034.308 |  |  |  |
| slope | Sphericity Assumed | 93795.000 | 2 | 46897.500 | 2.683 | .086 | .161 |
|  | Greenhouse-Geisser | 93795.000 | 1.674 | 56014.346 | 2.683 | .097 | .161 |
|  | Huynh-Feldt | 93795.000 | 1.876 | 50009.113 | 2.683 | .090 | .161 |
|  | Lower-bound | 93795.000 | 1.000 | 93795.000 | 2.683 | .124 | .161 |
| Error(slope) | Sphericity Assumed | 489357.583 | 28 | 17477.057 |  |  |  |
|  | Greenhouse-Geisser | 489357.583 | 23.443 | 20874.586 |  |  |  |
|  | Huynh-Feldt | 489357.583 | 26.258 | 18636.646 |  |  |  |
|  | Lower-bound | 489357.583 | 14.000 | 34954.113 |  |  |  |
| insole * slope | Sphericity Assumed | 4717.400 | 2 | 2358.700 | 1.250 | .302 | .082 |
|  | Greenhouse-Geisser | 4717.400 | 1.378 | 3423.825 | 1.250 | .294 | .082 |
|  | Huynh-Feldt | 4717.400 | 1.479 | 3189.754 | 1.250 | .296 | .082 |
|  | Lower-bound | 4717.400 | 1.000 | 4717.400 | 1.250 | .282 | .082 |
| Error(insole*slope) | Sphericity Assumed | 52824.683 | 28 | 1886.596 |  |  |  |
|  | Greenhouse-Geisser | 52824.683 | 19.289 | 2738.532 |  |  |  |
|  | Huynh-Feldt | 52824.683 | 20.705 | 2551.311 |  |  |  |
|  | Lower-bound | 52824.683 | 14.000 | 3773.192 |  |  |  |

**M3:**

|  | **Within-Subjects Factors** | | | |
| --- | --- | --- | --- | --- |
|  | Measure:MEASURE_1 | | | |
|  | insole | | slope | Dependent Variable |
| dimension1 | 1 | dimension2 | 1 | M3uphillASI |
|  |  |  | 2 | M3downhillASI |
|  |  |  | 3 | M3levelASI |
|  | 2 | dimension2 | 1 | M3uphillFI |
|  |  |  | 2 | M3downhillFI |
|  |  |  | 3 | M3levelFI |

| **Descriptive Statistics** | | | |
| --- | --- | --- | --- |
|  | Mean | Std. Deviation | N |
| M3uphillASI | 309.5000 | 156.28271 | 15 |
| M3downhillASI | 268.3000 | 158.67215 | 15 |
| M3levelASI | 383.5667 | 211.41672 | 15 |
| M3uphillFI | 293.1000 | 154.51378 | 15 |
| M3downhillFI | 218.5000 | 128.56946 | 15 |
| M3levelFI | 350.1667 | 176.90571 | 15 |

| **Tests of Within-Subjects Effects** | | | | | | | |
| --- | --- | --- | --- | --- | --- | --- | --- |
| Measure:MEASURE_1 | | | | | | | |
| Source | | Type III Sum of Squares | df | Mean Square | F | Sig. | Partial Eta Squared |
| insole | Sphericity Assumed | 24800.400 | 1 | 24800.400 | 8.197 | .013 | .369 |
|  | Greenhouse-Geisser | 24800.400 | 1.000 | 24800.400 | 8.197 | .013 | .369 |
|  | Huynh-Feldt | 24800.400 | 1.000 | 24800.400 | 8.197 | .013 | .369 |
|  | Lower-bound | 24800.400 | 1.000 | 24800.400 | 8.197 | .013 | .369 |
| Error(insole) | Sphericity Assumed | 42355.267 | 14 | 3025.376 |  |  |  |
|  | Greenhouse-Geisser | 42355.267 | 14.000 | 3025.376 |  |  |  |
|  | Huynh-Feldt | 42355.267 | 14.000 | 3025.376 |  |  |  |
|  | Lower-bound | 42355.267 | 14.000 | 3025.376 |  |  |  |
| slope | Sphericity Assumed | 228954.156 | 2 | 114477.078 | 6.136 | .006 | .305 |
|  | Greenhouse-Geisser | 228954.156 | 1.917 | 119446.989 | 6.136 | .007 | .305 |
|  | Huynh-Feldt | 228954.156 | 2.000 | 114477.078 | 6.136 | .006 | .305 |
|  | Lower-bound | 228954.156 | 1.000 | 228954.156 | 6.136 | .027 | .305 |
| Error(slope) | Sphericity Assumed | 522397.428 | 28 | 18657.051 |  |  |  |
|  | Greenhouse-Geisser | 522397.428 | 26.835 | 19467.029 |  |  |  |
|  | Huynh-Feldt | 522397.428 | 28.000 | 18657.051 |  |  |  |
|  | Lower-bound | 522397.428 | 14.000 | 37314.102 |  |  |  |
| insole * slope | Sphericity Assumed | 4183.800 | 2 | 2091.900 | 1.011 | .377 | .067 |
|  | Greenhouse-Geisser | 4183.800 | 1.623 | 2577.802 | 1.011 | .364 | .067 |
|  | Huynh-Feldt | 4183.800 | 1.805 | 2317.407 | 1.011 | .371 | .067 |
|  | Lower-bound | 4183.800 | 1.000 | 4183.800 | 1.011 | .332 | .067 |
| Error(insole*slope) | Sphericity Assumed | 57909.783 | 28 | 2068.207 |  |  |  |
|  | Greenhouse-Geisser | 57909.783 | 22.722 | 2548.605 |  |  |  |
|  | Huynh-Feldt | 57909.783 | 25.275 | 2291.160 |  |  |  |
|  | Lower-bound | 57909.783 | 14.000 | 4136.413 |  |  |  |

**M4:**

|  | **Within-Subjects Factors** | | | |
| --- | --- | --- | --- | --- |
|  | Measure:MEASURE_1 | | | |
|  | insole | | slope | Dependent Variable |
| dimension1 | 1 | dimension2 | 1 | M4uphillASI |
|  |  |  | 2 | M4downhillASI |
|  |  |  | 3 | M4levelASI |
|  | 2 | dimension2 | 1 | M4uphillFI |
|  |  |  | 2 | M4downhillFI |
|  |  |  | 3 | M4levelFI |

| **Descriptive Statistics** | | | |
| --- | --- | --- | --- |
|  | Mean | Std. Deviation | N |
| M4uphillASI | 207.3000 | 89.73071 | 15 |
| M4downhillASI | 151.9667 | 66.21946 | 15 |
| M4levelASI | 255.8000 | 97.96898 | 15 |
| M4uphillFI | 198.9667 | 85.10466 | 15 |
| M4downhillFI | 131.9667 | 45.79904 | 15 |
| M4levelFI | 226.3000 | 75.59828 | 15 |

| **Tests of Within-Subjects Effects** | | | | | | | |
| --- | --- | --- | --- | --- | --- | --- | --- |
| Measure:MEASURE_1 | | | | | | | |
| Source | | Type III Sum of Squares | df | Mean Square | F | Sig. | Partial Eta Squared |
| insole | Sphericity Assumed | 8361.736 | 1 | 8361.736 | 8.041 | .013 | .365 |
|  | Greenhouse-Geisser | 8361.736 | 1.000 | 8361.736 | 8.041 | .013 | .365 |
|  | Huynh-Feldt | 8361.736 | 1.000 | 8361.736 | 8.041 | .013 | .365 |
|  | Lower-bound | 8361.736 | 1.000 | 8361.736 | 8.041 | .013 | .365 |
| Error(insole) | Sphericity Assumed | 14558.556 | 14 | 1039.897 |  |  |  |
|  | Greenhouse-Geisser | 14558.556 | 14.000 | 1039.897 |  |  |  |
|  | Huynh-Feldt | 14558.556 | 14.000 | 1039.897 |  |  |  |
|  | Lower-bound | 14558.556 | 14.000 | 1039.897 |  |  |  |
| slope | Sphericity Assumed | 149965.417 | 2 | 74982.708 | 9.101 | .001 | .394 |
|  | Greenhouse-Geisser | 149965.417 | 1.856 | 80803.880 | 9.101 | .001 | .394 |
|  | Huynh-Feldt | 149965.417 | 2.000 | 74982.708 | 9.101 | .001 | .394 |
|  | Lower-bound | 149965.417 | 1.000 | 149965.417 | 9.101 | .009 | .394 |
| Error(slope) | Sphericity Assumed | 230687.167 | 28 | 8238.827 |  |  |  |
|  | Greenhouse-Geisser | 230687.167 | 25.983 | 8878.437 |  |  |  |
|  | Huynh-Feldt | 230687.167 | 28.000 | 8238.827 |  |  |  |
|  | Lower-bound | 230687.167 | 14.000 | 16477.655 |  |  |  |
| insole * slope | Sphericity Assumed | 1685.972 | 2 | 842.986 | 1.185 | .321 | .078 |
|  | Greenhouse-Geisser | 1685.972 | 1.563 | 1078.399 | 1.185 | .314 | .078 |
|  | Huynh-Feldt | 1685.972 | 1.725 | 977.470 | 1.185 | .317 | .078 |
|  | Lower-bound | 1685.972 | 1.000 | 1685.972 | 1.185 | .295 | .078 |
| Error(insole*slope) | Sphericity Assumed | 19925.111 | 28 | 711.611 |  |  |  |
|  | Greenhouse-Geisser | 19925.111 | 21.888 | 910.336 |  |  |  |
|  | Huynh-Feldt | 19925.111 | 24.148 | 825.137 |  |  |  |
|  | Lower-bound | 19925.111 | 14.000 | 1423.222 |  |  |  |

**M5:**

|  | **Within-Subjects Factors** | | | |
| --- | --- | --- | --- | --- |
|  | Measure:MEASURE_1 | | | |
|  | insole | | slope | Dependent Variable |
| dimension1 | 1 | dimension2 | 1 | M5uphillASI |
|  |  |  | 2 | M5downhillASI |
|  |  |  | 3 | M5levelASI |
|  | 2 | dimension2 | 1 | M5uphillFI |
|  |  |  | 2 | M5downhillFI |
|  |  |  | 3 | M5levelFI |

| **Descriptive Statistics** | | | |
| --- | --- | --- | --- |
|  | Mean | Std. Deviation | N |
| M5uphillASI | 147.1000 | 65.20002 | 15 |
| M5downhillASI | 112.5667 | 46.66211 | 15 |
| M5levelASI | 162.9333 | 48.25855 | 15 |
| M5uphillFI | 148.9000 | 73.37068 | 15 |
| M5downhillFI | 104.9667 | 39.22666 | 15 |
| M5levelFI | 154.3333 | 49.90515 | 15 |

| **Tests of Within-Subjects Effects** | | | | | | | |
| --- | --- | --- | --- | --- | --- | --- | --- |
| Measure:MEASURE_1 | | | | | | | |
| Source | | Type III Sum of Squares | df | Mean Square | F | Sig. | Partial Eta Squared |
| insole | Sphericity Assumed | 518.400 | 1 | 518.400 | 1.212 | .289 | .080 |
|  | Greenhouse-Geisser | 518.400 | 1.000 | 518.400 | 1.212 | .289 | .080 |
|  | Huynh-Feldt | 518.400 | 1.000 | 518.400 | 1.212 | .289 | .080 |
|  | Lower-bound | 518.400 | 1.000 | 518.400 | 1.212 | .289 | .080 |
| Error(insole) | Sphericity Assumed | 5987.517 | 14 | 427.680 |  |  |  |
|  | Greenhouse-Geisser | 5987.517 | 14.000 | 427.680 |  |  |  |
|  | Huynh-Feldt | 5987.517 | 14.000 | 427.680 |  |  |  |
|  | Lower-bound | 5987.517 | 14.000 | 427.680 |  |  |  |
| slope | Sphericity Assumed | 41390.067 | 2 | 20695.033 | 6.353 | .005 | .312 |
|  | Greenhouse-Geisser | 41390.067 | 1.591 | 26020.674 | 6.353 | .010 | .312 |
|  | Huynh-Feldt | 41390.067 | 1.762 | 23496.131 | 6.353 | .008 | .312 |
|  | Lower-bound | 41390.067 | 1.000 | 41390.067 | 6.353 | .024 | .312 |
| Error(slope) | Sphericity Assumed | 91204.600 | 28 | 3257.307 |  |  |  |
|  | Greenhouse-Geisser | 91204.600 | 22.269 | 4095.540 |  |  |  |
|  | Huynh-Feldt | 91204.600 | 24.662 | 3698.188 |  |  |  |
|  | Lower-bound | 91204.600 | 14.000 | 6514.614 |  |  |  |
| insole * slope | Sphericity Assumed | 493.800 | 2 | 246.900 | .558 | .579 | .038 |
|  | Greenhouse-Geisser | 493.800 | 1.554 | 317.749 | .558 | .538 | .038 |
|  | Huynh-Feldt | 493.800 | 1.712 | 288.389 | .558 | .553 | .038 |
|  | Lower-bound | 493.800 | 1.000 | 493.800 | .558 | .467 | .038 |
| Error(insole*slope) | Sphericity Assumed | 12386.533 | 28 | 442.376 |  |  |  |
|  | Greenhouse-Geisser | 12386.533 | 21.757 | 569.318 |  |  |  |
|  | Huynh-Feldt | 12386.533 | 23.972 | 516.712 |  |  |  |
|  | Lower-bound | 12386.533 | 14.000 | 884.752 |  |  |  |

**MF:**

|  | **Within-Subjects Factors** | | | |
| --- | --- | --- | --- | --- |
|  | Measure:MEASURE_1 | | | |
|  | insole | | slope | Dependent Variable |
| dimension1 | 1 | dimension2 | 1 | MFuphillASI |
|  |  |  | 2 | MFdownhillASI |
|  |  |  | 3 | MFlevelASI |
|  | 2 | dimension2 | 1 | MFuphillFI |
|  |  |  | 2 | MFdownhillFI |
|  |  |  | 3 | MFlevelFI |

| **Descriptive Statistics** | | | |
| --- | --- | --- | --- |
|  | Mean | Std. Deviation | N |
| MFuphillASI | 135.5667 | 64.15157 | 15 |
| MFdownhillASI | 120.4333 | 40.20921 | 15 |
| MFlevelASI | 115.3333 | 31.81344 | 15 |
| MFuphillFI | 140.4000 | 56.48774 | 15 |
| MFdownhillFI | 104.9667 | 34.37268 | 15 |
| MFlevelFI | 121.6333 | 36.73528 | 15 |

| **Tests of Within-Subjects Effects** | | | | | | | |
| --- | --- | --- | --- | --- | --- | --- | --- |
| Measure:MEASURE_1 | | | | | | | |
| Source | | Type III Sum of Squares | df | Mean Square | F | Sig. | Partial Eta Squared |
| insole | Sphericity Assumed | 46.944 | 1 | 46.944 | .048 | .829 | .003 |
|  | Greenhouse-Geisser | 46.944 | 1.000 | 46.944 | .048 | .829 | .003 |
|  | Huynh-Feldt | 46.944 | 1.000 | 46.944 | .048 | .829 | .003 |
|  | Lower-bound | 46.944 | 1.000 | 46.944 | .048 | .829 | .003 |
| Error(insole) | Sphericity Assumed | 13596.139 | 14 | 971.153 |  |  |  |
|  | Greenhouse-Geisser | 13596.139 | 14.000 | 971.153 |  |  |  |
|  | Huynh-Feldt | 13596.139 | 14.000 | 971.153 |  |  |  |
|  | Lower-bound | 13596.139 | 14.000 | 971.153 |  |  |  |
| slope | Sphericity Assumed | 10529.439 | 2 | 5264.719 | 2.694 | .085 | .161 |
|  | Greenhouse-Geisser | 10529.439 | 1.274 | 8264.078 | 2.694 | .112 | .161 |
|  | Huynh-Feldt | 10529.439 | 1.345 | 7830.632 | 2.694 | .109 | .161 |
|  | Lower-bound | 10529.439 | 1.000 | 10529.439 | 2.694 | .123 | .161 |
| Error(slope) | Sphericity Assumed | 54724.228 | 28 | 1954.437 |  |  |  |
|  | Greenhouse-Geisser | 54724.228 | 17.838 | 3067.897 |  |  |  |
|  | Huynh-Feldt | 54724.228 | 18.825 | 2906.988 |  |  |  |
|  | Lower-bound | 54724.228 | 14.000 | 3908.873 |  |  |  |
| insole * slope | Sphericity Assumed | 2220.072 | 2 | 1110.036 | 1.235 | .306 | .081 |
|  | Greenhouse-Geisser | 2220.072 | 1.426 | 1557.123 | 1.235 | .298 | .081 |
|  | Huynh-Feldt | 2220.072 | 1.542 | 1439.974 | 1.235 | .301 | .081 |
|  | Lower-bound | 2220.072 | 1.000 | 2220.072 | 1.235 | .285 | .081 |
| Error(insole*slope) | Sphericity Assumed | 25170.094 | 28 | 898.932 |  |  |  |
|  | Greenhouse-Geisser | 25170.094 | 19.961 | 1260.993 |  |  |  |
|  | Huynh-Feldt | 25170.094 | 21.584 | 1166.123 |  |  |  |
|  | Lower-bound | 25170.094 | 14.000 | 1797.864 |  |  |  |

**MH:**

|  | **Within-Subjects Factors** | | | |
| --- | --- | --- | --- | --- |
|  | Measure:MEASURE_1 | | | |
|  | insole | | slope | Dependent Variable |
| dimension1 | 1 | dimension2 | 1 | MHuphillASI |
|  |  |  | 2 | MHdownhillASI |
|  |  |  | 3 | MHlevelASI |
|  | 2 | dimension2 | 1 | MHuphillFI |
|  |  |  | 2 | MHdownhillFI |
|  |  |  | 3 | MHlevelFI |

| **Descriptive Statistics** | | | |
| --- | --- | --- | --- |
|  | Mean | Std. Deviation | N |
| MHuphillASI | 144.3333 | 64.36161 | 15 |
| MHdownhillASI | 167.9000 | 114.23225 | 15 |
| MHlevelASI | 163.7000 | 72.61144 | 15 |
| MHuphillFI | 167.3333 | 60.15328 | 15 |
| MHdownhillFI | 222.1333 | 134.17173 | 15 |
| MHlevelFI | 192.9667 | 62.98284 | 15 |

| **Tests of Within-Subjects Effects** | | | | | | | |
| --- | --- | --- | --- | --- | --- | --- | --- |
| Measure:MEASURE_1 | | | | | | | |
| Source | | Type III Sum of Squares | df | Mean Square | F | Sig. | Partial Eta Squared |
| insole | Sphericity Assumed | 28355.625 | 1 | 28355.625 | 26.351 | .000 | .653 |
|  | Greenhouse-Geisser | 28355.625 | 1.000 | 28355.625 | 26.351 | .000 | .653 |
|  | Huynh-Feldt | 28355.625 | 1.000 | 28355.625 | 26.351 | .000 | .653 |
|  | Lower-bound | 28355.625 | 1.000 | 28355.625 | 26.351 | .000 | .653 |
| Error(insole) | Sphericity Assumed | 15065.083 | 14 | 1076.077 |  |  |  |
|  | Greenhouse-Geisser | 15065.083 | 14.000 | 1076.077 |  |  |  |
|  | Huynh-Feldt | 15065.083 | 14.000 | 1076.077 |  |  |  |
|  | Lower-bound | 15065.083 | 14.000 | 1076.077 |  |  |  |
| slope | Sphericity Assumed | 23199.172 | 2 | 11599.586 | 1.891 | .170 | .119 |
|  | Greenhouse-Geisser | 23199.172 | 1.257 | 18450.592 | 1.891 | .187 | .119 |
|  | Huynh-Feldt | 23199.172 | 1.323 | 17533.190 | 1.891 | .185 | .119 |
|  | Lower-bound | 23199.172 | 1.000 | 23199.172 | 1.891 | .191 | .119 |
| Error(slope) | Sphericity Assumed | 171743.494 | 28 | 6133.696 |  |  |  |
|  | Greenhouse-Geisser | 171743.494 | 17.603 | 9756.411 |  |  |  |
|  | Huynh-Feldt | 171743.494 | 18.524 | 9271.302 |  |  |  |
|  | Lower-bound | 171743.494 | 14.000 | 12267.392 |  |  |  |
| insole * slope | Sphericity Assumed | 4095.317 | 2 | 2047.658 | 5.860 | .007 | .295 |
|  | Greenhouse-Geisser | 4095.317 | 1.680 | 2437.339 | 5.860 | .012 | .295 |
|  | Huynh-Feldt | 4095.317 | 1.883 | 2174.373 | 5.860 | .009 | .295 |
|  | Lower-bound | 4095.317 | 1.000 | 4095.317 | 5.860 | .030 | .295 |
| Error(insole*slope) | Sphericity Assumed | 9784.350 | 28 | 349.441 |  |  |  |
|  | Greenhouse-Geisser | 9784.350 | 23.523 | 415.942 |  |  |  |
|  | Huynh-Feldt | 9784.350 | 26.368 | 371.065 |  |  |  |
|  | Lower-bound | 9784.350 | 14.000 | 698.882 |  |  |  |

**LH:**

|  | **Within-Subjects Factors** | | | |
| --- | --- | --- | --- | --- |
|  | Measure:MEASURE_1 | | | |
|  | insole | | slope | Dependent Variable |
| dimension1 | 1 | dimension2 | 1 | LHuphillASI |
|  |  |  | 2 | LHdownhillASI |
|  |  |  | 3 | LHlevelASI |
|  | 2 | dimension2 | 1 | LHuphillFI |
|  |  |  | 2 | LHdownhillFI |
|  |  |  | 3 | LHlevelFI |

| **Descriptive Statistics** | | | |
| --- | --- | --- | --- |
|  | Mean | Std. Deviation | N |
| LHuphillASI | 111.9667 | 49.12356 | 15 |
| LHdownhillASI | 158.6000 | 99.01014 | 15 |
| LHlevelASI | 159.2000 | 90.45378 | 15 |
| LHuphillFI | 138.8000 | 71.66734 | 15 |
| LHdownhillFI | 181.9000 | 103.10245 | 15 |
| LHlevelFI | 169.4000 | 76.13526 | 15 |

| **Tests of Within-Subjects Effects** | | | | | | | |
| --- | --- | --- | --- | --- | --- | --- | --- |
| Measure:MEASURE_1 | | | | | | | |
| Source | | Type III Sum of Squares | df | Mean Square | F | Sig. | Partial Eta Squared |
| insole | Sphericity Assumed | 9100.278 | 1 | 9100.278 | 8.131 | .013 | .367 |
|  | Greenhouse-Geisser | 9100.278 | 1.000 | 9100.278 | 8.131 | .013 | .367 |
|  | Huynh-Feldt | 9100.278 | 1.000 | 9100.278 | 8.131 | .013 | .367 |
|  | Lower-bound | 9100.278 | 1.000 | 9100.278 | 8.131 | .013 | .367 |
| Error(insole) | Sphericity Assumed | 15668.222 | 14 | 1119.159 |  |  |  |
|  | Greenhouse-Geisser | 15668.222 | 14.000 | 1119.159 |  |  |  |
|  | Huynh-Feldt | 15668.222 | 14.000 | 1119.159 |  |  |  |
|  | Lower-bound | 15668.222 | 14.000 | 1119.159 |  |  |  |
| slope | Sphericity Assumed | 35629.272 | 2 | 17814.636 | 4.093 | .028 | .226 |
|  | Greenhouse-Geisser | 35629.272 | 1.658 | 21493.532 | 4.093 | .037 | .226 |
|  | Huynh-Feldt | 35629.272 | 1.853 | 19232.273 | 4.093 | .031 | .226 |
|  | Lower-bound | 35629.272 | 1.000 | 35629.272 | 4.093 | .063 | .226 |
| Error(slope) | Sphericity Assumed | 121867.728 | 28 | 4352.419 |  |  |  |
|  | Greenhouse-Geisser | 121867.728 | 23.207 | 5251.236 |  |  |  |
|  | Huynh-Feldt | 121867.728 | 25.936 | 4698.772 |  |  |  |
|  | Lower-bound | 121867.728 | 14.000 | 8704.838 |  |  |  |
| insole * slope | Sphericity Assumed | 1151.906 | 2 | 575.953 | .405 | .671 | .028 |
|  | Greenhouse-Geisser | 1151.906 | 1.806 | 637.826 | .405 | .651 | .028 |
|  | Huynh-Feldt | 1151.906 | 2.000 | 575.953 | .405 | .671 | .028 |
|  | Lower-bound | 1151.906 | 1.000 | 1151.906 | .405 | .535 | .028 |
| Error(insole*slope) | Sphericity Assumed | 39814.094 | 28 | 1421.932 |  |  |  |
|  | Greenhouse-Geisser | 39814.094 | 25.284 | 1574.687 |  |  |  |
|  | Huynh-Feldt | 39814.094 | 28.000 | 1421.932 |  |  |  |
|  | Lower-bound | 39814.094 | 14.000 | 2843.864 |  |  |  |
