# Supplementary material for: The arch support insoles show benefits to people with flatfoot on stance time, cadence, plantar pressure and contact area
Source: PLoS One. 2020 Aug 20;15(8):e0237382. doi: 10.1371/journal.pone.0237382 (PMC7446821; doi:10.1371/journal.pone.0237382)
Supplement: S1 Data — (ZIP) [file pone.0237382.s001.zip › peak pressure-Simple main effects.docx]

**ＢＴ**

**Uphill:**

| **Within-Subjects Factors** | | |
| --- | --- | --- |
| Measure:MEASURE_1 | | |
| factor1 | | Dependent Variable |
| dimension1 | 1 | BTuphillASI |
|  | 2 | BTuphillFI |

| **Descriptive Statistics** | | | |
| --- | --- | --- | --- |
|  | Mean | Std. Deviation | N |
| BTuphillASI | 407.7000 | 249.94312 | 15 |
| BTuphillFI | 294.1000 | 171.99171 | 15 |

| **Tests of Within-Subjects Effects** | | | | | | | |
| --- | --- | --- | --- | --- | --- | --- | --- |
| Measure:MEASURE_1 | | | | | | | |
| Source | | Type III Sum of Squares | df | Mean Square | F | Sig. | Partial Eta Squared |
| factor1 | Sphericity Assumed | 96787.200 | 1 | 96787.200 | 14.325 | .002 | .506 |
|  | Greenhouse-Geisser | 96787.200 | 1.000 | 96787.200 | 14.325 | .002 | .506 |
|  | Huynh-Feldt | 96787.200 | 1.000 | 96787.200 | 14.325 | .002 | .506 |
|  | Lower-bound | 96787.200 | 1.000 | 96787.200 | 14.325 | .002 | .506 |
| Error(factor1) | Sphericity Assumed | 94594.300 | 14 | 6756.736 |  |  |  |
|  | Greenhouse-Geisser | 94594.300 | 14.000 | 6756.736 |  |  |  |
|  | Huynh-Feldt | 94594.300 | 14.000 | 6756.736 |  |  |  |
|  | Lower-bound | 94594.300 | 14.000 | 6756.736 |  |  |  |

**Downhill:**

| **Within-Subjects Factors** | | |
| --- | --- | --- |
| Measure:MEASURE_1 | | |
| factor1 | | Dependent Variable |
| dimension1 | 1 | BTdownhillASI |
|  | 2 | BTdownhillFI |

| **Descriptive Statistics** | | | |
| --- | --- | --- | --- |
|  | Mean | Std. Deviation | N |
| BTdownhillASI | 322.7333 | 133.89373 | 15 |
| BTdownhillFI | 284.8333 | 111.10463 | 15 |

| **Tests of Within-Subjects Effects** | | | | | | | |
| --- | --- | --- | --- | --- | --- | --- | --- |
| Measure:MEASURE_1 | | | | | | | |
| Source | | Type III Sum of Squares | df | Mean Square | F | Sig. | Partial Eta Squared |
| factor1 | Sphericity Assumed | 10773.075 | 1 | 10773.075 | 2.348 | .148 | .144 |
|  | Greenhouse-Geisser | 10773.075 | 1.000 | 10773.075 | 2.348 | .148 | .144 |
|  | Huynh-Feldt | 10773.075 | 1.000 | 10773.075 | 2.348 | .148 | .144 |
|  | Lower-bound | 10773.075 | 1.000 | 10773.075 | 2.348 | .148 | .144 |
| Error(factor1) | Sphericity Assumed | 64226.800 | 14 | 4587.629 |  |  |  |
|  | Greenhouse-Geisser | 64226.800 | 14.000 | 4587.629 |  |  |  |
|  | Huynh-Feldt | 64226.800 | 14.000 | 4587.629 |  |  |  |
|  | Lower-bound | 64226.800 | 14.000 | 4587.629 |  |  |  |

**Level:**

| **Within-Subjects Factors** | | |
| --- | --- | --- |
| Measure:MEASURE_1 | | |
| factor1 | | Dependent Variable |
| dimension1 | 1 | BTlevelASI |
|  | 2 | BTlevelFI |

| **Descriptive Statistics** | | | |
| --- | --- | --- | --- |
|  | Mean | Std. Deviation | N |
| BTlevelASI | 226.4000 | 80.64610 | 15 |
| BTlevelFI | 171.2000 | 74.67711 | 15 |

| **Tests of Within-Subjects Effects** | | | | | | | |
| --- | --- | --- | --- | --- | --- | --- | --- |
| Measure:MEASURE_1 | | | | | | | |
| Source | | Type III Sum of Squares | df | Mean Square | F | Sig. | Partial Eta Squared |
| factor1 | Sphericity Assumed | 22852.800 | 1 | 22852.800 | 7.091 | .019 | .336 |
|  | Greenhouse-Geisser | 22852.800 | 1.000 | 22852.800 | 7.091 | .019 | .336 |
|  | Huynh-Feldt | 22852.800 | 1.000 | 22852.800 | 7.091 | .019 | .336 |
|  | Lower-bound | 22852.800 | 1.000 | 22852.800 | 7.091 | .019 | .336 |
| Error(factor1) | Sphericity Assumed | 45115.950 | 14 | 3222.568 |  |  |  |
|  | Greenhouse-Geisser | 45115.950 | 14.000 | 3222.568 |  |  |  |
|  | Huynh-Feldt | 45115.950 | 14.000 | 3222.568 |  |  |  |
|  | Lower-bound | 45115.950 | 14.000 | 3222.568 |  |  |  |

**Ａｒｃｈ　ｓｕｐｐｏｒｔ　ｉｎｓｏｌｅ：**

| **Within-Subjects Factors** | | | | | | | | |  |  |  |  |  |  |  |  |  |  |  |  |  |  |  |
| --- | --- | --- | --- | --- | --- | --- | --- | --- | --- | --- | --- | --- | --- | --- | --- | --- | --- | --- | --- | --- | --- | --- | --- |
| Measure:MEASURE_1 | | | | | | | | |  |  |  |  |  |  |  |  |  |  |  |  |  |  |  |
| factor1 | | | Dependent Variable | | | | | |  |  |  |  |  |  |  |  |  |  |  |  |  |  |  |
| dimension1 | 1 | | BTuphillASI | | | | | |  |  |  |  |  |  |  |  |  |  |  |  |  |  |  |
|  | 2 | | BTdownhillASI | | | | | |  |  |  |  |  |  |  |  |  |  |  |  |  |  |  |
|  | 3 | | BTlevelASI | | | | | |  |  |  |  |  |  |  |  |  |  |  |  |  |  |  |
| **Descriptive Statistics** | | | | | | | | | | | | | | | |  |  |  |  |  |  |  |  |
|  | | | | | | | Mean | | | Std. Deviation | | | N | | |  |  |  |  |  |  |  |  |
| BTuphillASI | | | | | | | 407.7000 | | | 249.94312 | | | 15 | | |  |  |  |  |  |  |  |  |
| BTdownhillASI | | | | | | | 322.7333 | | | 133.89373 | | | 15 | | |  |  |  |  |  |  |  |  |
| BTlevelASI | | | | | | | 226.4000 | | | 80.64610 | | | 15 | | |  |  |  |  |  |  |  |  |
| **Tests of Within-Subjects Effects** | | | | | | | | | | | | | | | | | | | | | | | |
| Measure:MEASURE_1 | | | | | | | | | | | | | | | | | | | | | | | |
| Source | | | | | | | | | | | Type III Sum of Squares | | | | df | | | Mean Square | | F | Sig. | | Partial Eta Squared |
| factor1 | | | | | Sphericity Assumed | | | | | | 246845.678 | | | | 2 | | | 123422.839 | | 7.473 | .003 | | .348 |
|  |  |  |  |  | Greenhouse-Geisser | | | | | | 246845.678 | | | | 1.569 | | | 157287.882 | | 7.473 | .006 | | .348 |
|  |  |  |  |  | Huynh-Feldt | | | | | | 246845.678 | | | | 1.733 | | | 142447.845 | | 7.473 | .004 | | .348 |
|  |  |  |  |  | Lower-bound | | | | | | 246845.678 | | | | 1.000 | | | 246845.678 | | 7.473 | .016 | | .348 |
| Error(factor1) | | | | | Sphericity Assumed | | | | | | 462465.489 | | | | 28 | | | 16516.625 | |  |  | |  |
|  |  |  |  |  | Greenhouse-Geisser | | | | | | 462465.489 | | | | 21.971 | | | 21048.494 | |  |  | |  |
|  |  |  |  |  | Huynh-Feldt | | | | | | 462465.489 | | | | 24.260 | | | 19062.579 | |  |  | |  |
|  |  |  |  |  | Lower-bound | | | | | | 462465.489 | | | | 14.000 | | | 33033.249 | |  |  | |  |
| **Pairwise Comparisons** | | | | | | | | | | | | | | | | | | | | | |  |  |
| Measure:MEASURE_1 | | | | | | | | | | | | | | | | | | | | | |  |  |
| (I) factor1 | | | | (J) factor1 | | | | Mean Difference (I-J) | | | | Std. Error | | Sig.^a^ | | | 95% Confidence Interval for Difference^a^ | | | | |  |  |
|  |  |  |  |  |  |  |  |  |  |  |  |  |  |  |  |  | Lower Bound | | Upper Bound | | |  |  |
| dimension1 | | 1 | | dimension2 | | 2 | | 84.967 | | | | 50.182 | | .338 | | | -51.415 | | 221.348 | | |  |  |
|  |  |  |  |  |  | 3 | | 181.300^*^ | | | | 54.818 | | .016 | | | 32.318 | | 330.282 | | |  |  |
|  |  | 2 | | dimension2 | | 1 | | -84.967 | | | | 50.182 | | .338 | | | -221.348 | | 51.415 | | |  |  |
|  |  |  |  |  |  | 3 | | 96.333^*^ | | | | 32.915 | | .033 | | | 6.877 | | 185.789 | | |  |  |
|  |  | 3 | | dimension2 | | 1 | | -181.300^*^ | | | | 54.818 | | .016 | | | -330.282 | | -32.318 | | |  |  |
|  |  |  |  |  |  | 2 | | -96.333^*^ | | | | 32.915 | | .033 | | | -185.789 | | -6.877 | | |  |  |
| Based on estimated marginal means | | | | | | | | | | | | | | | | | | | | | |  |  |
| a. Adjustment for multiple comparisons: Bonferroni. | | | | | | | | | | | | | | | | | | | | | |  |  |
| *. The mean difference is significant at the .05 level. | | | | | | | | | | | | | | | | | | | | | |  |  |

**Ｆｌａｔ　ｉｎｓｏｌｅ：**

| **Within-Subjects Factors** | | | | | | | | |  |  |  |  |  |  |  |  |  |  |  |  |  |  |  |
| --- | --- | --- | --- | --- | --- | --- | --- | --- | --- | --- | --- | --- | --- | --- | --- | --- | --- | --- | --- | --- | --- | --- | --- |
| Measure:MEASURE_1 | | | | | | | | |  |  |  |  |  |  |  |  |  |  |  |  |  |  |  |
| factor1 | | | Dependent Variable | | | | | |  |  |  |  |  |  |  |  |  |  |  |  |  |  |  |
| dimension1 | 1 | | BTuphillFI | | | | | |  |  |  |  |  |  |  |  |  |  |  |  |  |  |  |
|  | 2 | | BTdownhillFI | | | | | |  |  |  |  |  |  |  |  |  |  |  |  |  |  |  |
|  | 3 | | BTlevelFI | | | | | |  |  |  |  |  |  |  |  |  |  |  |  |  |  |  |
| **Descriptive Statistics** | | | | | | | | | | | | | | |  |  |  |  |  |  |  |  |  |
|  | | | | | Mean | | | | | Std. Deviation | | | N | |  |  |  |  |  |  |  |  |  |
| BTuphillFI | | | | | 294.1000 | | | | | 171.99171 | | | 15 | |  |  |  |  |  |  |  |  |  |
| BTdownhillFI | | | | | 284.8333 | | | | | 111.10463 | | | 15 | |  |  |  |  |  |  |  |  |  |
| BTlevelFI | | | | | 171.2000 | | | | | 74.67711 | | | 15 | |  |  |  |  |  |  |  |  |  |
| **Tests of Within-Subjects Effects** | | | | | | | | | | | | | | | | | | | | | | | |
| Measure:MEASURE_1 | | | | | | | | | | | | | | | | | | | | | | | |
| Source | | | | | | | | | | | Type III Sum of Squares | | | | | df | | Mean Square | | F | Sig. | | Partial Eta Squared |
| factor1 | | | | | | Sphericity Assumed | | | | | 140514.078 | | | | | 2 | | 70257.039 | | 6.268 | .006 | | .309 |
|  |  |  |  |  |  | Greenhouse-Geisser | | | | | 140514.078 | | | | | 1.656 | | 84828.379 | | 6.268 | .009 | | .309 |
|  |  |  |  |  |  | Huynh-Feldt | | | | | 140514.078 | | | | | 1.851 | | 75916.308 | | 6.268 | .007 | | .309 |
|  |  |  |  |  |  | Lower-bound | | | | | 140514.078 | | | | | 1.000 | | 140514.078 | | 6.268 | .025 | | .309 |
| Error(factor1) | | | | | | Sphericity Assumed | | | | | 313830.256 | | | | | 28 | | 11208.223 | |  |  | |  |
|  |  |  |  |  |  | Greenhouse-Geisser | | | | | 313830.256 | | | | | 23.190 | | 13532.814 | |  |  | |  |
|  |  |  |  |  |  | Huynh-Feldt | | | | | 313830.256 | | | | | 25.913 | | 12111.056 | |  |  | |  |
|  |  |  |  |  |  | Lower-bound | | | | | 313830.256 | | | | | 14.000 | | 22416.447 | |  |  | |  |
| **Pairwise Comparisons** | | | | | | | | | | | | | | | | | | | | | |  |  |
| Measure:MEASURE_1 | | | | | | | | | | | | | | | | | | | | | |  |  |
| (I) factor1 | | | | (J) factor1 | | | | Mean Difference (I-J) | | | | Std. Error | | Sig.^a^ | | | 95% Confidence Interval for Difference^a^ | | | | |  |  |
|  |  |  |  |  |  |  |  |  |  |  |  |  |  |  |  |  | Lower Bound | | Upper Bound | | |  |  |
| dimension1 | | 1 | | dimension2 | | | 2 | 9.267 | | | | 45.945 | | 1.000 | | | -115.600 | | 134.133 | | |  |  |
|  |  |  |  |  |  |  | 3 | 122.900^*^ | | | | 37.893 | | .018 | | | 19.916 | | 225.884 | | |  |  |
|  |  | 2 | | dimension2 | | | 1 | -9.267 | | | | 45.945 | | 1.000 | | | -134.133 | | 115.600 | | |  |  |
|  |  |  |  |  |  |  | 3 | 113.633^*^ | | | | 30.602 | | .007 | | | 30.464 | | 196.802 | | |  |  |
|  |  | 3 | | dimension2 | | | 1 | -122.900^*^ | | | | 37.893 | | .018 | | | -225.884 | | -19.916 | | |  |  |
|  |  |  |  |  |  |  | 2 | -113.633^*^ | | | | 30.602 | | .007 | | | -196.802 | | -30.464 | | |  |  |
| Based on estimated marginal means | | | | | | | | | | | | | | | | | | | | | |  |  |
| a. Adjustment for multiple comparisons: Bonferroni. | | | | | | | | | | | | | | | | | | | | | |  |  |
| *. The mean difference is significant at the .05 level. | | | | | | | | | | | | | | | | | | | | | |  |  |

**ＭＨ**

**ｕｐｈｉｌｌ：**

| **Within-Subjects Factors** | | |
| --- | --- | --- |
| Measure:MEASURE_1 | | |
| factor1 | | Dependent Variable |
| dimension1 | 1 | MHuphillASI |
|  | 2 | MHuphillFI |

| **Descriptive Statistics** | | | |
| --- | --- | --- | --- |
|  | Mean | Std. Deviation | N |
| MHuphillASI | 144.3333 | 64.36161 | 15 |
| MHuphillFI | 167.3333 | 60.15328 | 15 |

| **Tests of Within-Subjects Effects** | | | | | | | |
| --- | --- | --- | --- | --- | --- | --- | --- |
| Measure:MEASURE_1 | | | | | | | |
| Source | | Type III Sum of Squares | df | Mean Square | F | Sig. | Partial Eta Squared |
| factor1 | Sphericity Assumed | 3967.500 | 1 | 3967.500 | 13.068 | .003 | .483 |
|  | Greenhouse-Geisser | 3967.500 | 1.000 | 3967.500 | 13.068 | .003 | .483 |
|  | Huynh-Feldt | 3967.500 | 1.000 | 3967.500 | 13.068 | .003 | .483 |
|  | Lower-bound | 3967.500 | 1.000 | 3967.500 | 13.068 | .003 | .483 |
| Error(factor1) | Sphericity Assumed | 4250.500 | 14 | 303.607 |  |  |  |
|  | Greenhouse-Geisser | 4250.500 | 14.000 | 303.607 |  |  |  |
|  | Huynh-Feldt | 4250.500 | 14.000 | 303.607 |  |  |  |
|  | Lower-bound | 4250.500 | 14.000 | 303.607 |  |  |  |

**ｄｏｗｎｈｉｌｌ：**

| **Within-Subjects Factors** | | |
| --- | --- | --- |
| Measure:MEASURE_1 | | |
| factor1 | | Dependent Variable |
| dimension1 | 1 | MHdownhillASI |
|  | 2 | MHdownhillFI |

| **Descriptive Statistics** | | | | | | |  |  |  |  |  |
| --- | --- | --- | --- | --- | --- | --- | --- | --- | --- | --- | --- |
|  | | Mean | Std. Deviation | | N | |  |  |  |  |  |
| MHdownhillASI | | 167.9000 | 114.23225 | | 15 | |  |  |  |  |  |
| MHdownhillFI | | 222.1333 | 134.17173 | | 15 | |  |  |  |  |  |
| **Tests of Within-Subjects Effects** | | | | | | | | | | | |
| Measure:MEASURE_1 | | | | | | | | | | | |
| Source | | | | Type III Sum of Squares | | df | | Mean Square | F | Sig. | Partial Eta Squared |
| factor1 | Sphericity Assumed | | | 22059.408 | | 1 | | 22059.408 | 47.913 | .000 | .774 |
|  | Greenhouse-Geisser | | | 22059.408 | | 1.000 | | 22059.408 | 47.913 | .000 | .774 |
|  | Huynh-Feldt | | | 22059.408 | | 1.000 | | 22059.408 | 47.913 | .000 | .774 |
|  | Lower-bound | | | 22059.408 | | 1.000 | | 22059.408 | 47.913 | .000 | .774 |
| Error(factor1) | Sphericity Assumed | | | 6445.717 | | 14 | | 460.408 |  |  |  |
|  | Greenhouse-Geisser | | | 6445.717 | | 14.000 | | 460.408 |  |  |  |
|  | Huynh-Feldt | | | 6445.717 | | 14.000 | | 460.408 |  |  |  |
|  | Lower-bound | | | 6445.717 | | 14.000 | | 460.408 |  |  |  |

**ｌｅｖｅｌ：**

| **Within-Subjects Factors** | | | | | |  |  |  |  |  |  |  |  |  |
| --- | --- | --- | --- | --- | --- | --- | --- | --- | --- | --- | --- | --- | --- | --- |
| Measure:MEASURE_1 | | | | | |  |  |  |  |  |  |  |  |  |
| factor1 | | Dependent Variable | | | |  |  |  |  |  |  |  |  |  |
| dimension1 | 1 | MHlevelASI | | | |  |  |  |  |  |  |  |  |  |
|  | 2 | MHlevelFI | | | |  |  |  |  |  |  |  |  |  |
| **Descriptive Statistics** | | | | | | | | |  |  |  |  |  |  |
|  | | | Mean | | Std. Deviation | | | N |  |  |  |  |  |  |
| MHlevelASI | | | 163.7000 | | 72.61144 | | | 15 |  |  |  |  |  |  |
| MHlevelFI | | | 192.9667 | | 62.98284 | | | 15 |  |  |  |  |  |  |
| **Tests of Within-Subjects Effects** | | | | | | | | | | | | | | |
| Measure:MEASURE_1 | | | | | | | | | | | | | | |
| Source | | | | | | | Type III Sum of Squares | | | df | Mean Square | F | Sig. | Partial Eta Squared |
| factor1 | | | | Sphericity Assumed | | | 6424.033 | | | 1 | 6424.033 | 6.354 | .024 | .312 |
|  |  |  |  | Greenhouse-Geisser | | | 6424.033 | | | 1.000 | 6424.033 | 6.354 | .024 | .312 |
|  |  |  |  | Huynh-Feldt | | | 6424.033 | | | 1.000 | 6424.033 | 6.354 | .024 | .312 |
|  |  |  |  | Lower-bound | | | 6424.033 | | | 1.000 | 6424.033 | 6.354 | .024 | .312 |
| Error(factor1) | | | | Sphericity Assumed | | | 14153.217 | | | 14 | 1010.944 |  |  |  |
|  |  |  |  | Greenhouse-Geisser | | | 14153.217 | | | 14.000 | 1010.944 |  |  |  |
|  |  |  |  | Huynh-Feldt | | | 14153.217 | | | 14.000 | 1010.944 |  |  |  |
|  |  |  |  | Lower-bound | | | 14153.217 | | | 14.000 | 1010.944 |  |  |  |

**Ａｒｃｈ　ｓｕｐｐｏｒｔ　ｉｎｓｏｌｅ：**

| **Within-Subjects Factors** | | | | | | | | |  |  |  |  |  |  |  |  |  |  |  |  |  |  |  |
| --- | --- | --- | --- | --- | --- | --- | --- | --- | --- | --- | --- | --- | --- | --- | --- | --- | --- | --- | --- | --- | --- | --- | --- |
| Measure:MEASURE_1 | | | | | | | | |  |  |  |  |  |  |  |  |  |  |  |  |  |  |  |
| factor1 | | | Dependent Variable | | | | | |  |  |  |  |  |  |  |  |  |  |  |  |  |  |  |
| dimension1 | 1 | | MHuphillASI | | | | | |  |  |  |  |  |  |  |  |  |  |  |  |  |  |  |
|  | 2 | | MHdownhillASI | | | | | |  |  |  |  |  |  |  |  |  |  |  |  |  |  |  |
|  | 3 | | MHlevelASI | | | | | |  |  |  |  |  |  |  |  |  |  |  |  |  |  |  |
| **Descriptive Statistics** | | | | | | | | | | | | | | | |  |  |  |  |  |  |  |  |
|  | | | | | | | Mean | | | Std. Deviation | | | N | | |  |  |  |  |  |  |  |  |
| MHuphillASI | | | | | | | 144.3333 | | | 64.36161 | | | 15 | | |  |  |  |  |  |  |  |  |
| MHdownhillASI | | | | | | | 167.9000 | | | 114.23225 | | | 15 | | |  |  |  |  |  |  |  |  |
| MHlevelASI | | | | | | | 163.7000 | | | 72.61144 | | | 15 | | |  |  |  |  |  |  |  |  |
| **Tests of Within-Subjects Effects** | | | | | | | | | | | | | | | | | | | | | | | |
| Measure:MEASURE_1 | | | | | | | | | | | | | | | | | | | | | | | |
| Source | | | | | | | | | | | Type III Sum of Squares | | | | df | | | Mean Square | | F | Sig. | | Partial Eta Squared |
| factor1 | | | | | Sphericity Assumed | | | | | | 4740.478 | | | | 2 | | | 2370.239 | | .735 | .489 | | .050 |
|  |  |  |  |  | Greenhouse-Geisser | | | | | | 4740.478 | | | | 1.384 | | | 3425.955 | | .735 | .445 | | .050 |
|  |  |  |  |  | Huynh-Feldt | | | | | | 4740.478 | | | | 1.487 | | | 3188.800 | | .735 | .453 | | .050 |
|  |  |  |  |  | Lower-bound | | | | | | 4740.478 | | | | 1.000 | | | 4740.478 | | .735 | .406 | | .050 |
| Error(factor1) | | | | | Sphericity Assumed | | | | | | 90336.856 | | | | 28 | | | 3226.316 | |  |  | |  |
|  |  |  |  |  | Greenhouse-Geisser | | | | | | 90336.856 | | | | 19.372 | | | 4663.333 | |  |  | |  |
|  |  |  |  |  | Huynh-Feldt | | | | | | 90336.856 | | | | 20.812 | | | 4340.523 | |  |  | |  |
|  |  |  |  |  | Lower-bound | | | | | | 90336.856 | | | | 14.000 | | | 6452.633 | |  |  | |  |
| **Pairwise Comparisons** | | | | | | | | | | | | | | | | | | | | | |  |  |
| Measure:MEASURE_1 | | | | | | | | | | | | | | | | | | | | | |  |  |
| (I) factor1 | | | | (J) factor1 | | | | Mean Difference (I-J) | | | | Std. Error | | Sig.^a^ | | | 95% Confidence Interval for Difference^a^ | | | | |  |  |
|  |  |  |  |  |  |  |  |  |  |  |  |  |  |  |  |  | Lower Bound | | Upper Bound | | |  |  |
| dimension1 | | 1 | | dimension2 | | 2 | | -23.567 | | | | 22.600 | | .944 | | | -84.988 | | 37.855 | | |  |  |
|  |  |  |  |  |  | 3 | | -19.367 | | | | 12.298 | | .413 | | | -52.790 | | 14.057 | | |  |  |
|  |  | 2 | | dimension2 | | 1 | | 23.567 | | | | 22.600 | | .944 | | | -37.855 | | 84.988 | | |  |  |
|  |  |  |  |  |  | 3 | | 4.200 | | | | 25.070 | | 1.000 | | | -63.934 | | 72.334 | | |  |  |
|  |  | 3 | | dimension2 | | 1 | | 19.367 | | | | 12.298 | | .413 | | | -14.057 | | 52.790 | | |  |  |
|  |  |  |  |  |  | 2 | | -4.200 | | | | 25.070 | | 1.000 | | | -72.334 | | 63.934 | | |  |  |
| Based on estimated marginal means | | | | | | | | | | | | | | | | | | | | | |  |  |
| a. Adjustment for multiple comparisons: Bonferroni. | | | | | | | | | | | | | | | | | | | | | |  |  |

**Ｆｌａｔ　ｉｎｓｏｌｅ：**

| **Within-Subjects Factors** | | | | | | | | |  |  |  |  |  |  |  |  |  |  |  |  |  |  |  |
| --- | --- | --- | --- | --- | --- | --- | --- | --- | --- | --- | --- | --- | --- | --- | --- | --- | --- | --- | --- | --- | --- | --- | --- |
| Measure:MEASURE_1 | | | | | | | | |  |  |  |  |  |  |  |  |  |  |  |  |  |  |  |
| factor1 | | | Dependent Variable | | | | | |  |  |  |  |  |  |  |  |  |  |  |  |  |  |  |
| dimension1 | 1 | | MHuphillFI | | | | | |  |  |  |  |  |  |  |  |  |  |  |  |  |  |  |
|  | 2 | | MHdownhillFI | | | | | |  |  |  |  |  |  |  |  |  |  |  |  |  |  |  |
|  | 3 | | MHlevelFI | | | | | |  |  |  |  |  |  |  |  |  |  |  |  |  |  |  |
| **Descriptive Statistics** | | | | | | | | | | | | | | | |  |  |  |  |  |  |  |  |
|  | | | | | | Mean | | | | Std. Deviation | | | N | | |  |  |  |  |  |  |  |  |
| MHuphillFI | | | | | | 167.3333 | | | | 60.15328 | | | 15 | | |  |  |  |  |  |  |  |  |
| MHdownhillFI | | | | | | 222.1333 | | | | 134.17173 | | | 15 | | |  |  |  |  |  |  |  |  |
| MHlevelFI | | | | | | 192.9667 | | | | 62.98284 | | | 15 | | |  |  |  |  |  |  |  |  |
| **Tests of Within-Subjects Effects** | | | | | | | | | | | | | | | | | | | | | | | |
| Measure:MEASURE_1 | | | | | | | | | | | | | | | | | | | | | | | |
| Source | | | | | | | | | | | Type III Sum of Squares | | | | df | | | Mean Square | | F | Sig. | | Partial Eta Squared |
| factor1 | | | | | Sphericity Assumed | | | | | | 22554.011 | | | | 2 | | | 11277.006 | | 3.463 | .045 | | .198 |
|  |  |  |  |  | Greenhouse-Geisser | | | | | | 22554.011 | | | | 1.200 | | | 18794.087 | | 3.463 | .074 | | .198 |
|  |  |  |  |  | Huynh-Feldt | | | | | | 22554.011 | | | | 1.250 | | | 18042.130 | | 3.463 | .072 | | .198 |
|  |  |  |  |  | Lower-bound | | | | | | 22554.011 | | | | 1.000 | | | 22554.011 | | 3.463 | .084 | | .198 |
| Error(factor1) | | | | | Sphericity Assumed | | | | | | 91190.989 | | | | 28 | | | 3256.821 | |  |  | |  |
|  |  |  |  |  | Greenhouse-Geisser | | | | | | 91190.989 | | | | 16.801 | | | 5427.769 | |  |  | |  |
|  |  |  |  |  | Huynh-Feldt | | | | | | 91190.989 | | | | 17.501 | | | 5210.602 | |  |  | |  |
|  |  |  |  |  | Lower-bound | | | | | | 91190.989 | | | | 14.000 | | | 6513.642 | |  |  | |  |
| **Pairwise Comparisons** | | | | | | | | | | | | | | | | | | | | | |  |  |
| Measure:MEASURE_1 | | | | | | | | | | | | | | | | | | | | | |  |  |
| (I) factor1 | | | | (J) factor1 | | | | Mean Difference (I-J) | | | | Std. Error | | Sig.^a^ | | | 95% Confidence Interval for Difference^a^ | | | | |  |  |
|  |  |  |  |  |  |  |  |  |  |  |  |  |  |  |  |  | Lower Bound | | Upper Bound | | |  |  |
| dimension1 | | 1 | | dimension2 | | | 2 | -54.800 | | | | 24.882 | | .135 | | | -122.424 | | 12.824 | | |  |  |
|  |  |  |  |  |  |  | 3 | -25.633^*^ | | | | 8.934 | | .037 | | | -49.914 | | -1.353 | | |  |  |
|  |  | 2 | | dimension2 | | | 1 | 54.800 | | | | 24.882 | | .135 | | | -12.824 | | 122.424 | | |  |  |
|  |  |  |  |  |  |  | 3 | 29.167 | | | | 24.572 | | .765 | | | -37.613 | | 95.947 | | |  |  |
|  |  | 3 | | dimension2 | | | 1 | 25.633^*^ | | | | 8.934 | | .037 | | | 1.353 | | 49.914 | | |  |  |
|  |  |  |  |  |  |  | 2 | -29.167 | | | | 24.572 | | .765 | | | -95.947 | | 37.613 | | |  |  |
| Based on estimated marginal means | | | | | | | | | | | | | | | | | | | | | |  |  |
| a. Adjustment for multiple comparisons: Bonferroni. | | | | | | | | | | | | | | | | | | | | | |  |  |
| *. The mean difference is significant at the .05 level. | | | | | | | | | | | | | | | | | | | | | |  |  |
