# Supplementary material for: The arch support insoles show benefits to people with flatfoot on stance time, cadence, plantar pressure and contact area
Source: PLoS One. 2020 Aug 20;15(8):e0237382. doi: 10.1371/journal.pone.0237382 (PMC7446821; doi:10.1371/journal.pone.0237382)
Supplement: S1 Data — (ZIP) [file pone.0237382.s001.zip › stance time-Interaction and main effects.docx]

|  | **Within-Subjects Factors** | | | |
| --- | --- | --- | --- | --- |
|  | Measure:MEASURE_1 | | | |
|  | insole | | slope | Dependent Variable |
| dimension1 | 1 | dimension2 | 1 | uphillASI |
|  |  |  | 2 | downhillASI |
|  |  |  | 3 | levelASI |
|  | 2 | dimension2 | 1 | uphillFI |
|  |  |  | 2 | downhillFI |
|  |  |  | 3 | levelFI |

| **Descriptive Statistics** | | | |
| --- | --- | --- | --- |
|  | Mean | Std. Deviation | N |
| uphillASI | .7303 | .10460 | 15 |
| downhillASI | .6507 | .07238 | 15 |
| levelASI | .7507 | .07794 | 15 |
| uphillFI | .7413 | .08863 | 15 |
| downhillFI | .6527 | .08194 | 15 |
| levelFI | .7857 | .06774 | 15 |

| **Tests of Within-Subjects Effects** | | | | | | | |
| --- | --- | --- | --- | --- | --- | --- | --- |
| Measure:MEASURE_1 | | | | | | | |
| Source | | Type III Sum of Squares | df | Mean Square | F | Sig. | Partial Eta Squared |
| insole | Sphericity Assumed | .006 | 1 | .006 | 11.536 | .004 | .452 |
|  | Greenhouse-Geisser | .006 | 1.000 | .006 | 11.536 | .004 | .452 |
|  | Huynh-Feldt | .006 | 1.000 | .006 | 11.536 | .004 | .452 |
|  | Lower-bound | .006 | 1.000 | .006 | 11.536 | .004 | .452 |
| Error(insole) | Sphericity Assumed | .007 | 14 | .000 |  |  |  |
|  | Greenhouse-Geisser | .007 | 14.000 | .000 |  |  |  |
|  | Huynh-Feldt | .007 | 14.000 | .000 |  |  |  |
|  | Lower-bound | .007 | 14.000 | .000 |  |  |  |
| slope | Sphericity Assumed | .217 | 2 | .109 | 13.363 | .000 | .488 |
|  | Greenhouse-Geisser | .217 | 1.973 | .110 | 13.363 | .000 | .488 |
|  | Huynh-Feldt | .217 | 2.000 | .109 | 13.363 | .000 | .488 |
|  | Lower-bound | .217 | 1.000 | .217 | 13.363 | .003 | .488 |
| Error(slope) | Sphericity Assumed | .227 | 28 | .008 |  |  |  |
|  | Greenhouse-Geisser | .227 | 27.626 | .008 |  |  |  |
|  | Huynh-Feldt | .227 | 28.000 | .008 |  |  |  |
|  | Lower-bound | .227 | 14.000 | .016 |  |  |  |
| insole * slope | Sphericity Assumed | .004 | 2 | .002 | 3.333 | .050 | .192 |
|  | Greenhouse-Geisser | .004 | 1.985 | .002 | 3.333 | .051 | .192 |
|  | Huynh-Feldt | .004 | 2.000 | .002 | 3.333 | .050 | .192 |
|  | Lower-bound | .004 | 1.000 | .004 | 3.333 | .089 | .192 |
| Error(insole*slope) | Sphericity Assumed | .018 | 28 | .001 |  |  |  |
|  | Greenhouse-Geisser | .018 | 27.785 | .001 |  |  |  |
|  | Huynh-Feldt | .018 | 28.000 | .001 |  |  |  |
|  | Lower-bound | .018 | 14.000 | .001 |  |  |  |
