# Supplementary material for: The arch support insoles show benefits to people with flatfoot on stance time, cadence, plantar pressure and contact area
Source: PLoS One. 2020 Aug 20;15(8):e0237382. doi: 10.1371/journal.pone.0237382 (PMC7446821; doi:10.1371/journal.pone.0237382)
Supplement: S1 Data — (ZIP) [file pone.0237382.s001.zip › stance time-Simple main effects.docx]

| **Within-Subjects Factors** | | |
| --- | --- | --- |
| Measure:MEASURE_1 | | |
| factor1 | | Dependent Variable |
| dimension1 | 1 | uphillASI |
|  | 2 | uphillFI |

| **Tests of Within-Subjects Effects** | | | | | | |
| --- | --- | --- | --- | --- | --- | --- |
| Measure:MEASURE_1 | | | | | | |
| Source | | Type III Sum of Squares | df | Mean Square | F | Sig. |
| factor1 | Sphericity Assumed | .001 | 1 | .001 | 1.431 | .252 |
|  | Greenhouse-Geisser | .001 | 1.000 | .001 | 1.431 | .252 |
|  | Huynh-Feldt | .001 | 1.000 | .001 | 1.431 | .252 |
|  | Lower-bound | .001 | 1.000 | .001 | 1.431 | .252 |
| Error(factor1) | Sphericity Assumed | .009 | 14 | .001 |  |  |
|  | Greenhouse-Geisser | .009 | 14.000 | .001 |  |  |
|  | Huynh-Feldt | .009 | 14.000 | .001 |  |  |
|  | Lower-bound | .009 | 14.000 | .001 |  |  |

| **Within-Subjects Factors** | | | |  |  |  |  |  |  |
| --- | --- | --- | --- | --- | --- | --- | --- | --- | --- |
| Measure:MEASURE_1 | | | |  |  |  |  |  |  |
| factor1 | | Dependent Variable | |  |  |  |  |  |  |
| dimension1 | 1 | downhillASI | |  |  |  |  |  |  |
|  | 2 | downhillFI | |  |  |  |  |  |  |
| **Tests of Within-Subjects Effects** | | | | | | | | | |
| Measure:MEASURE_1 | | | | | | | | | |
| Source | | | | | Type III Sum of Squares | df | Mean Square | F | Sig. |
| factor1 | | | Sphericity Assumed | | 3.000E-5 | 1 | 3.000E-5 | .057 | .815 |
|  |  |  | Greenhouse-Geisser | | 3.000E-5 | 1.000 | 3.000E-5 | .057 | .815 |
|  |  |  | Huynh-Feldt | | 3.000E-5 | 1.000 | 3.000E-5 | .057 | .815 |
|  |  |  | Lower-bound | | 3.000E-5 | 1.000 | 3.000E-5 | .057 | .815 |
| Error(factor1) | | | Sphericity Assumed | | .007 | 14 | .001 |  |  |
|  |  |  | Greenhouse-Geisser | | .007 | 14.000 | .001 |  |  |
|  |  |  | Huynh-Feldt | | .007 | 14.000 | .001 |  |  |
|  |  |  | Lower-bound | | .007 | 14.000 | .001 |  |  |

| **Within-Subjects Factors** | | |
| --- | --- | --- |
| Measure:MEASURE_1 | | |
| factor1 | | Dependent Variable |
| dimension1 | 1 | levelASI |
|  | 2 | levelFI |

| **Tests of Within-Subjects Effects** | | | | | | |
| --- | --- | --- | --- | --- | --- | --- |
| Measure:MEASURE_1 | | | | | | |
| Source | | Type III Sum of Squares | df | Mean Square | F | Sig. |
| factor1 | Sphericity Assumed | .009 | 1 | .009 | 14.174 | .002 |
|  | Greenhouse-Geisser | .009 | 1.000 | .009 | 14.174 | .002 |
|  | Huynh-Feldt | .009 | 1.000 | .009 | 14.174 | .002 |
|  | Lower-bound | .009 | 1.000 | .009 | 14.174 | .002 |
| Error(factor1) | Sphericity Assumed | .009 | 14 | .001 |  |  |
|  | Greenhouse-Geisser | .009 | 14.000 | .001 |  |  |
|  | Huynh-Feldt | .009 | 14.000 | .001 |  |  |
|  | Lower-bound | .009 | 14.000 | .001 |  |  |

| **Within-Subjects Factors** | | | | | | | | | |  |  |  |  |  |  |  |  |  |  |  |  |  |  |
| --- | --- | --- | --- | --- | --- | --- | --- | --- | --- | --- | --- | --- | --- | --- | --- | --- | --- | --- | --- | --- | --- | --- | --- |
| Measure:MEASURE_1 | | | | | | | | | |  |  |  |  |  |  |  |  |  |  |  |  |  |  |
| factor1 | | | Dependent Variable | | | | | | |  |  |  |  |  |  |  |  |  |  |  |  |  |  |
| dimension1 | 1 | | uphillASI | | | | | | |  |  |  |  |  |  |  |  |  |  |  |  |  |  |
|  | 2 | | downhillASI | | | | | | |  |  |  |  |  |  |  |  |  |  |  |  |  |  |
|  | 3 | | levelASI | | | | | | |  |  |  |  |  |  |  |  |  |  |  |  |  |  |
| **Descriptive Statistics** | | | | | | | | | | | | | | |  |  |  |  |  |  |  |  |  |
|  | | | | | Mean | | | | Std. Deviation | | | | N | |  |  |  |  |  |  |  |  |  |
| uphillASI | | | | | .7303 | | | | .10460 | | | | 15 | |  |  |  |  |  |  |  |  |  |
| downhillASI | | | | | .6507 | | | | .07238 | | | | 15 | |  |  |  |  |  |  |  |  |  |
| levelASI | | | | | .7507 | | | | .07794 | | | | 15 | |  |  |  |  |  |  |  |  |  |
| **Tests of Within-Subjects Effects** | | | | | | | | | | | | | | | | | | | | | | | |
| Measure:MEASURE_1 | | | | | | | | | | | | | | | | | | | | | | | |
| Source | | | | | | | | | | | Type III Sum of Squares | | | | | df | | Mean Square | | F | Sig. | | Partial Eta Squared |
| factor1 | | | | | | Sphericity Assumed | | | | | .084 | | | | | 2 | | .042 | | 9.676 | .001 | | .409 |
|  |  |  |  |  |  | Greenhouse-Geisser | | | | | .084 | | | | | 1.932 | | .043 | | 9.676 | .001 | | .409 |
|  |  |  |  |  |  | Huynh-Feldt | | | | | .084 | | | | | 2.000 | | .042 | | 9.676 | .001 | | .409 |
|  |  |  |  |  |  | Lower-bound | | | | | .084 | | | | | 1.000 | | .084 | | 9.676 | .008 | | .409 |
| Error(factor1) | | | | | | Sphericity Assumed | | | | | .121 | | | | | 28 | | .004 | |  |  | |  |
|  |  |  |  |  |  | Greenhouse-Geisser | | | | | .121 | | | | | 27.052 | | .004 | |  |  | |  |
|  |  |  |  |  |  | Huynh-Feldt | | | | | .121 | | | | | 28.000 | | .004 | |  |  | |  |
|  |  |  |  |  |  | Lower-bound | | | | | .121 | | | | | 14.000 | | .009 | |  |  | |  |
| **Pairwise Comparisons** | | | | | | | | | | | | | | | | | | | | | |  |  |
| Measure:MEASURE_1 | | | | | | | | | | | | | | | | | | | | | |  |  |
| (I) factor1 | | | | (J) factor1 | | | | Mean Difference (I-J) | | | | Std. Error | | Sig.^a^ | | | 95% Confidence Interval for Difference^a^ | | | | |  |  |
|  |  |  |  |  |  |  |  |  |  |  |  |  |  |  |  |  | Lower Bound | | Upper Bound | | |  |  |
| dimension1 | | 1 | | dimension2 | | | 2 | .080^*^ | | | | .025 | | .019 | | | .012 | | .147 | | |  |  |
|  |  |  |  |  |  |  | 3 | -.020 | | | | .025 | | 1.000 | | | -.089 | | .049 | | |  |  |
|  |  | 2 | | dimension2 | | | 1 | -.080^*^ | | | | .025 | | .019 | | | -.147 | | -.012 | | |  |  |
|  |  |  |  |  |  |  | 3 | -.100^*^ | | | | .022 | | .001 | | | -.159 | | -.041 | | |  |  |
|  |  | 3 | | dimension2 | | | 1 | .020 | | | | .025 | | 1.000 | | | -.049 | | .089 | | |  |  |
|  |  |  |  |  |  |  | 2 | .100^*^ | | | | .022 | | .001 | | | .041 | | .159 | | |  |  |
| Based on estimated marginal means | | | | | | | | | | | | | | | | | | | | | |  |  |
| *. The mean difference is significant at the .05 level. | | | | | | | | | | | | | | | | | | | | | |  |  |
| a. Adjustment for multiple comparisons: Bonferroni. | | | | | | | | | | | | | | | | | | | | | |  |  |

| **Within-Subjects Factors** | | | | | | | | | |  |  |  |  |  |  |  |  |  |  |  |  |  |  |
| --- | --- | --- | --- | --- | --- | --- | --- | --- | --- | --- | --- | --- | --- | --- | --- | --- | --- | --- | --- | --- | --- | --- | --- |
| Measure:MEASURE_1 | | | | | | | | | |  |  |  |  |  |  |  |  |  |  |  |  |  |  |
| factor1 | | | Dependent Variable | | | | | | |  |  |  |  |  |  |  |  |  |  |  |  |  |  |
| dimension1 | 1 | | uphillFI | | | | | | |  |  |  |  |  |  |  |  |  |  |  |  |  |  |
|  | 2 | | downhillFI | | | | | | |  |  |  |  |  |  |  |  |  |  |  |  |  |  |
|  | 3 | | levelFI | | | | | | |  |  |  |  |  |  |  |  |  |  |  |  |  |  |
| **Descriptive Statistics** | | | | | | | | | | | | | |  |  |  |  |  |  |  |  |  |  |
|  | | | | | Mean | | | Std. Deviation | | | | N | |  |  |  |  |  |  |  |  |  |  |
| uphillFI | | | | | .7413 | | | .08863 | | | | 15 | |  |  |  |  |  |  |  |  |  |  |
| downhillFI | | | | | .6527 | | | .08194 | | | | 15 | |  |  |  |  |  |  |  |  |  |  |
| levelFI | | | | | .7857 | | | .06774 | | | | 15 | |  |  |  |  |  |  |  |  |  |  |
| **Tests of Within-Subjects Effects** | | | | | | | | | | | | | | | | | | | | | | | |
| Measure:MEASURE_1 | | | | | | | | | | | | | | | | | | | | | | | |
| Source | | | | | | | | | | | Type III Sum of Squares | | | | | df | | Mean Square | | F | Sig. | | Partial Eta Squared |
| factor1 | | | | | | Sphericity Assumed | | | | | .138 | | | | | 2 | | .069 | | 15.477 | .000 | | .525 |
|  |  |  |  |  |  | Greenhouse-Geisser | | | | | .138 | | | | | 1.997 | | .069 | | 15.477 | .000 | | .525 |
|  |  |  |  |  |  | Huynh-Feldt | | | | | .138 | | | | | 2.000 | | .069 | | 15.477 | .000 | | .525 |
|  |  |  |  |  |  | Lower-bound | | | | | .138 | | | | | 1.000 | | .138 | | 15.477 | .001 | | .525 |
| Error(factor1) | | | | | | Sphericity Assumed | | | | | .124 | | | | | 28 | | .004 | |  |  | |  |
|  |  |  |  |  |  | Greenhouse-Geisser | | | | | .124 | | | | | 27.953 | | .004 | |  |  | |  |
|  |  |  |  |  |  | Huynh-Feldt | | | | | .124 | | | | | 28.000 | | .004 | |  |  | |  |
|  |  |  |  |  |  | Lower-bound | | | | | .124 | | | | | 14.000 | | .009 | |  |  | |  |
| **Pairwise Comparisons** | | | | | | | | | | | | | | | | | | | | | |  |  |
| Measure:MEASURE_1 | | | | | | | | | | | | | | | | | | | | | |  |  |
| (I) factor1 | | | | (J) factor1 | | | | | Mean Difference (I-J) | | | | Std. Error | | Sig.^a^ | | 95% Confidence Interval for Difference^a^ | | | | |  |  |
|  |  |  |  |  |  |  |  |  |  |  |  |  |  |  |  |  | Lower Bound | | Upper Bound | | |  |  |
| dimension1 | | 1 | | dimension2 | | | 2 | | .089^*^ | | | | .025 | | .009 | | .021 | | .156 | | |  |  |
|  |  |  |  |  |  |  | 3 | | -.044 | | | | .024 | | .260 | | -.110 | | .021 | | |  |  |
|  |  | 2 | | dimension2 | | | 1 | | -.089^*^ | | | | .025 | | .009 | | -.156 | | -.021 | | |  |  |
|  |  |  |  |  |  |  | 3 | | -.133^*^ | | | | .024 | | .000 | | -.199 | | -.067 | | |  |  |
|  |  | 3 | | dimension2 | | | 1 | | .044 | | | | .024 | | .260 | | -.021 | | .110 | | |  |  |
|  |  |  |  |  |  |  | 2 | | .133^*^ | | | | .024 | | .000 | | .067 | | .199 | | |  |  |
| Based on estimated marginal means | | | | | | | | | | | | | | | | | | | | | |  |  |
| *. The mean difference is significant at the .05 level. | | | | | | | | | | | | | | | | | | | | | |  |  |
| a. Adjustment for multiple comparisons: Bonferroni. | | | | | | | | | | | | | | | | | | | | | |  |  |
