# Supplementary material for: The arch support insoles show benefits to people with flatfoot on stance time, cadence, plantar pressure and contact area
Source: PLoS One. 2020 Aug 20;15(8):e0237382. doi: 10.1371/journal.pone.0237382 (PMC7446821; doi:10.1371/journal.pone.0237382)
Supplement: Attachment — Submitted filename: PONE-D-20-05316 - Comments.docx [file pone.0237382.s002.docx]

General comments:

I would like to thank the editor for inviting me to review the manuscript entitled “The arch support insoles show benefits to people with flatfoot on gait process time, plantar pressure and contact area”. This study compared various plantar pressure measurements while walking on three slopes and while wearing two different types of insoles. Please find below my comments, which may aid the authors with submitting a revised version of their manuscript.

- I think that the authors made a great attempt to link their variables of interest to measures of importance to people with flatfeet. However, the authors attempted to link simple plantar pressure variables to mobility and walking benefits without describing what is meant by these terms. I would recommend to clearly state what is meant by the terms “mobility” and “benefits” and how improvements in mobility look like.
- One of the authors’ main conclusions, namely that increased midfoot area relative to total area in the arch-support insole is representative of the restoring of a “normal” arch is, in my opinion, an incorrect statement. If the loaded midfoot area increases, it means that more of the midfoot is in contact with the ground. This would mean that the foot is actually in a more flattened position. I would strongly recommend revising the statements made upon these findings.
- Lastly, I would recommend removing figure 1, as it does not provide valuable information to the understanding of the methods of this paper. I would also recommend providing all units within tables 1-4 instead of including them in the table headings. The units in table 1 are unclear. Also, the p-values and Cohen’s d effect sizes should be provided for all comparisons in all tables.

Specific comments:

Line 43: In the introduction of the main text, you have not mentioned any previous study investigating increased pressure at the hip, knee, or ankle joints in people with flat feet; but you talk about it in the background section of the abstract. Please either introduce the observed changes at the hip, knee, and ankle joints from the previous studies in the introduction of the main text or delete this statement from the background section of the abstract. Also, I would strongly recommend to change the wording from “pressure on the lower back, hip, …” to “loading on the lower back, hip, …”.

Line 44: “… and risk serious damage to these joints …”. You have not provided any evidence for this statement in the main text. Please either remove this statement from the abstract or elaborate on this and provide references in the main text.

Line 51: Please consider rephrasing the sentence to “The significance level α was set to 0.05.”

Lines 52-58: Please include Cohen’s d effect sizes for the reported comparisons. Also, please reorder the results section so that you always mention the same insole first in your comparisons. This would make it easier for the reader to follow your findings.

Line 60: It is unclear how your reported findings “provide benefits in mobility”. You cannot make this statement with the results that you presented in the above section unless you clearly demonstrate how these variables are related to benefits in mobility.

Line 74: “… suffers from rigid the flatfoot …”: remove “the” from the sentence

Line 80: Please include a reference at the end of the sentence.

Lines 83-84: This sentence suggests that flatfoot can result in damage to internal organs and the brain. This is incorrect. Please remove this statement from the manuscript.

Line 87: Please include a reference at the end of the sentence.

Line 91: I think using the term “cure rate” is inappropriate for two reasons: 1) leg internal rotation and leg length discrepancy is not a “disease” and therefore cannot be “cured”, 2) wearing insoles does not “cure” anything, it may reduce excessive leg internal rotation or offset leg length discrepancy. Please replace the term “cure rate” with a more appropriate term.

Line 95: the peak vertical ground reaction force is not the propulsion force. They are probably related to each other but they are not the same. Therefore, please remove “(i.e., propulsion)” from the sentence.

Lines 102-104: this sentence is the same as the sentence before that. Please remove this sentence and include its references to the previous sentence.

Lines 106-108: this is not a hypothesis. Clearly state how wearing the arch-insole will affect your variables of interest.

Line 112: remove “with” from the sentence.

Line 116: Consider rephrasing the sentence to: “A priori sample size calculation was performed using GPower (Company, City, Country), …”.

Line 118: Why were the means and standard deviations for the medial-lateral COP used to perform the sample size calculations if these are not the variables of interest in your study?

Line 122: the plural of “index” is “indices”

Line 123: “… were considered to have the flatfoot …”. Remove “the” from the sentence.

Lines 125-127: the review board that approved this study is not affiliated with any institution of any of the authors. Could you please elaborate on that?

Line 134-135: Please consider rephrasing the sentence to: “The insole consists of 960 individual pressure measuring sensors.”

Lines 142-143: Please consider rephrasing the sentence to: “Each subject performed a 3 min warm-up period on the treadmill at self-selected pace.”

Lines 143-147: In one sentence you say that participants walked on all slopes one after the other with a 6 min break in between slopes. In the next sentence, however, you state that walking on different slopes was performed on different days. Those are contradicting statements. Which one is true?

Line 151: How was the hardness measured exactly. More information is needed.

Line 158: “gait process time”. This variable is typically referred to as “stance time” or “ground contact time”. Please consider changing this throughout the manuscript to not confuse the reader.

Line 161: remove “each” from the sentence.

Line 177: Please include a reference for how Cohen’s d effect sizes were calculated.

Line 185: was there a difference in stance times for the other slopes? Even if not, please include a sentence stating so and also include the p-value and effect size for the comparison.

Lines 190-212: Please change the order of reporting your comparisons. Sometimes you first mention the arch-support insole and other times you first mention the flat insole. Please pick one insole that you will always mention first in your comparisons. This would confuse the reader less.

Lines 210-211: please include your p-values and ES right after the slope condition.

Lines 217-219: you said that the arch-support insole shortened the time of each gait cycle. This is incorrect. A gait cycle goes from the heel strike of one foot to the heel strike of the same foot. Your findings showed, however, that the stance times were altered. Please revise.

Line 218: you state that mobility was improved. None of your variables of interest is a measure for mobility. At least you did not make good enough of a case that a variable you investigated actually represents mobility. Please provide more evidence that stance times are a legitimate measure for mobility or remove this statement from the paper.

Line 220: you state that the arch-support insole absorbed the shock associated with the foot contacting the ground. Again, this is a pure speculation and you have not provided any evidence that this is the case. Please provide the evidence or delete this statement from the manuscript.

Lines 238-239: I do not understand this sentence. Please elaborate what you want to say here.

Lines 244-245: It is only a speculation that wearing arch-support insoles will “restore a normal, elastic arch in people with flatfoot, …”. Please clearly state that this is only a speculation or include a reference that has shown this in the past. Also, what is a “normal” arch?

Lines 247-249: You cannot make this statement. You have not demonstrated why reducing peak pressures would be a benefit for people with flatfoot. Please make a better case of how you link peak pressure to substantial benefits in people or remove this statement.

Lines 256-258: How does wearing an arch-support insole restore a “normal”, elastic arch? What do you speculate is the mechanism behind it? Again, what is “normal”? Perhaps, you should not use this term.

Lines 258-265: You have not assessed arch deformation in any way in this study; yet, you discuss the windlass mechanism and how the arch may or may not collapse with these insoles. I would strongly recommend highlighting that these are speculations, only, or remove the entire paragraph from the manuscript.

Lines 269: how would the forefoot play a crucial role in the braking during downhill walking if all participants were striking with the heel? The forefoot will mainly still be in the air during the braking period of the stance phase. Therefore, the forefoot cannot contribute to the braking at all. Again, this is a speculation because you have not assessed forefoot motion. Please remove this statement from the paper.

Lines 276-278: If the area of the midfoot increases relative to the total area, then the foot is flatter compared to when the midfoot area is lower compared to the total area. This is also called the arch index (Cavanagh, 1987). Your conclusion, therefore, does not make sense. Please revise.

Line 281: remove “make” from the sentence.

Lines 293-294: you cannot make the statement that wearing the arch-support insole provided many benefits in mobility. You have not provided clear reasoning as to why this would be the case. Please remove this statement from the paper.
