# Supplementary material for: The arch support insoles show benefits to people with flatfoot on stance time, cadence, plantar pressure and contact area
Source: PLoS One. 2020 Aug 20;15(8):e0237382. doi: 10.1371/journal.pone.0237382 (PMC7446821; doi:10.1371/journal.pone.0237382)
Supplement: Attachment — Submitted filename: Response to Reviewers #2 ped 200526.docx [file pone.0237382.s003.docx]

**RESPOND to Reviewer #2:**

1.The statistics need to change. There was a comparison of insole condition across different levels of incline. The results seemed to indicate that the variables that were different across insole conditions seemed to be dependent on the level of incline. With this observation in mind, the researchers should re-run the statistics on each dependent variable and instead of conducting multiple uncorrected t-tests, they should conduct a two way repeated measures ANOVA with factors INCLINE (uphill, level, downhill) and INSOLE (flat, arch support). This will indicate if there is an interaction effect for the measured pressure and gait variables.
**RESPOND:**

**Thank you for your comment. We considered the reviewer’s comment to conduct a two-way repeated measures ANOVA with factors. The purpose of this study was mainly to compare the differences between the arch-support insole and flat insole in each of three slopes, respectively. Therefore, some of significance of the slopes did not discussed in the manuscript. Please refer to lines 178, 189-240 of the methods section, results and tables 2-4.**

2. Why did you restrict you sample to only females, particularly when the condition seems to be more common in males? Please provide an explanation why there wasn’t any males.
**RESPOND:**

**Thank you for your comment. We currently only recruit females this time in case of some homogeneity issues. We will further recruit male participants and compare the difference between males and females.**

3. Please provide an explanation as to why your static arch index was representative of flatfoot. My understanding is that flat foot would have something to do with the proportion of the foot that is or is not in contact with the ground in a loaded or unloaded condition. What does the narrowest and widest part of the foot have to do with the arch collapsing or per planus?
   **RESPOND:**

**Thank you for your comment. There are around 30 methods to evaluate the flatfoot (Banwell et. al, 2018; Cavanagh & Rodgers, 1987). There are two conditions of measuring the static arch index. One is in a body-weight loaded condition; another is in an body-weight unloaded condition. The current study used the measurement in a body-weight loaded condition. The static arch index is related to the ratio between the smallest width of the midfoot and the largest width of the metatarsal head area (Gill, Lewis, and DeSilva, 2014). This is also called the Chippaux and Smirakarc index (CSI) developed by Chippaux and Smirakarch. The CSI is correlated with skeletal measures of arch height such as the navicular height. People with flatfoot tend to have lower arch height during a stance based on a footprint. Then, the narrowest part of the foot will increase and change the CSI. The CSI had reliability and validity to measure and define flat foot populations (Gill, Lewis, and DeSilva, 2014).**

**Reference:**

**Banwell HA, Paris ME, Mackintosh S, Williams CM. Paediatric flexible flat foot: how are we measuring it and are we getting it right? A systematic review. Journal of Foot & Ankle Research. 2018; 11(1): 21-33. doi:10.1186/s13047-018-0264-3**

**Cavanagh PR, Rodgers MM. The arch index: a useful measure from footprints. Journal of Biomechanics. 1987; 20(5): 547–51. doi: 10.1016/0021-9290(87)90255-7**

**Gill SV, Lewis CL, DeSilva JM. Arch Height Mediation of Obesity-Related Walking in Adults: Contributors to Physical Activity Limitations. Physiology Journal. 2014; Article ID 821482** [**doi: 10.1155/2014/821482**](https://doi.org/10.1155/2014/821482)

4. Where was the pressure insole placed relative to the insoles (i.e., underneath or above)? Please provide an explanation why you used on placement method over the other.
**RESPOND:**

**Thank you for your comment. Wireless pressure insole with the F-Scan sensor (Model #3000E Tekscan, Inc., Boston, MA, USA) were placed above either the tested flat insole or arch-support insole of each foot to detect participant’s plantar pressure. The pressure insole was placed between the tested insole and foot to measure the contact pressure variables of the foot on the tested insole. If the pressure insole is placed underneath the tested insole, the contact pressure variables would be the tested insole rather than the foot and they may be affected by many other factors such as the thickness and the shape of the insole itself.**

5. How much of the results were due to hardness/stiffness of the insole versus it having arch support or not? For example, there was a difference in midsole hardness in the arch support condition and this condition also presented with an increase contact area. The same results applies to the forefoot area such that the harder condition showed increase contact area.
**RESPOND:**

**Thank you for your comment. The arch-support insole provide support in the midsole with greater hardness compared with the flat insole. However, the current study cannot show how much of the results were due to hardness of the insole versus it having arch support or not. It has been claimed in the limitation. Please refer to lines 301-303.**

6. Please change “gait process time” to stance time that would be more consistent with the literature.
**RESPOND:**

**Thanks for the comment. We have revised this throughout the manuscript. Please refer to lines 48, 54, 67, 107, 161, 163, 189, 190, 192, 193, 194, 250, 252, 254 and 258.**

7. Please present the data across the dominant and non-dominant leg. If in one leg there was higher peak pressure and lower peak pressure in the other, this would average out to no change in pressure. I don’t think your averaging method avoids discrepancies.
Please incorporate these changes and adjust the discussion accordingly. Once those comments are addressed, I will be able to provide more feedback on the minor, or major, comments that remain.

**RESPOND:**

**Thanks for the comment. We provided the data of dominant and non-dominant leg. Please refer to table 2-4. In addition, foot pressure was collected by F-Scan sensor (Model #3000E Tekscan, Inc., Boston, MA, USA) in the stance phase for each foot. In a stance phase, the F-Scan sensor counted the support foot pressure, while the swing foot pressure did not be counted. We averaged data of dominant and non-dominant leg measured during stance phases.**
