# Supplementary material for: The arch support insoles show benefits to people with flatfoot on stance time, cadence, plantar pressure and contact area
Source: PLoS One. 2020 Aug 20;15(8):e0237382. doi: 10.1371/journal.pone.0237382 (PMC7446821; doi:10.1371/journal.pone.0237382)
Supplement: Attachment — Submitted filename: RESPOND to Reviewer #2 ped 200707.docx [file pone.0237382.s004.docx]

Reviewer #2: Thank you to the authors for making some of the requested changed. The authors will still need to address the following comments:

1. Please provide the rationale as to why only females were recruited in the text of the manuscript under the methods section.

**RESPOND:**

**Thank you for your comment. Previous study has estimated the prevalence of mild and severe cases of the flatfoot to be 16.2% among males and 11.7% among females which was close to each other in gender (Tenenbaum et al., 2013). Nevertheless, females are more likely to suffer from risks of lower extremity injuries in running (Alomonroeder & Benson, 2017) and lack of flexible foot/shank coupling coordination compared to males (Noghondar & Yazdi, 2017). We have provided the rational in the introduction. Please refer to lines 83-86 .**

**References:**

(1) **Tenenbaum S, Hershkovich O, Gordon B, Bruck N, Thein R, Derazne E, et al. Flexible pes planus in adolescents: body mass index, body height, and gender--an epidemiological study. Foot & Ankle International. 2013; 34(6): 811-817. doi: 10.1177/1071100712472327**
(2) **Alomonroeder TG, Benson LC. Sex differences in lower extremity kinematics and patellofemoral kinetics during running. J Sport Sci. 2017; 35(16): 1575-1581. doi:** [**10.1080/02640414.2016.1225972**](https://doi.org/10.1080/02640414.2016.1225972)
(3) **Noghondar FA, Yazdi NK. Assessment of patterns and variability in lower extremity coordination between genders with different shoe insole stiffness during jump-landing tasks. Human Movement. 2017; 18(1): 37–43. doi: 10.1515/humo-2017-0002**
2. You did not provide a sufficient statement of the hardness of the insoles as a limitation. You need to be specific. You should be stating that the arch support insole did have higher hardness in the midsole and the flat insole was harder in the forefoot and the heel. This difference in stiffness could be a confounding variable to the results in the study and therefore, you cannot conclude that the differences were due to differences in hardness or the presence vs absence of an arch support

**RESPOND:**

**Thank you for your comment. We have added the review’s comment regarding the hardness of the insoles in the limitation section. Please refer to lines 330-334.**

3. Your purpose statement reads “the purpose of this study was to investigate the effects of the arch-support insoles on stance time, cadence, peak pressure, and contact area of the foot with the ground while walking uphill, downhill, and on a level surface, respectively.” In the discussion, you did not provide much explanation of the interaction effects that were found. You need to perform a post-hoc analysis on the interaction effects to determine the location of the interaction effect. For example, stance time had an interaction effect across insole and slope. It appears that this difference occurred in level walking, but not uphill and downhill walking. This needs to be determined with the post-hoc analysis and then an interpretation should be provided in the discussion. Please complete this type of analysis and interpretation for each interaction effect.

**RESPOND:**

**Thank you for your comment. We have performed a *post-hoc* analysis on the interaction effects and provided information regarding the interaction effects in the discussion section. Please refer to lines 273-275, 289-291, 301-303, and 305-307.**
